# A review of *Leucosigma* Druce, 1908: a newly discovered case of fern-feeding and descriptions of three new species (Lepidoptera, Noctuidae)

**DOI:** 10.3897/zookeys.788.21222

**Published:** 2018-10-08

**Authors:** Paul Z. Goldstein, Daniel H. Janzen, Benjamin Proshek, Tanya Dapkey, Winnie Hallwachs

**Affiliations:** 1 Systematic Entomology Laboratory, USDA, National Museum of Natural History, P.O. Box 37012, MRC 168, Washington, DC 20013-7012, USA; 2 Department of Biology, University of Pennsylvania, Philadelphia, PA 19104, USA

**Keywords:** *
Chytonidia
*, Costa Rica, DNA barcode, *
Leucosigma
*, pteridivore

## Abstract

*Chytonidia* Schaus, 1914, is one of two noctuine genera originally described by Schaus that includes species recently found to feed on fern foliage (Pteridophyta) as larvae. By examining museum specimens, including type material and reared specimens accompanied by DNA barcode data, *Chytonidia* Schaus, 1914, **syn. n.** is synonymized with *Leucosigma* Druce, 1908, all currently recognized species are re-described, including males of three species described from female holotypes, and three new species are described: *Leucosigmasolisae* Goldstein, **sp. n.**, *Leucosigmapoolei* Goldstein, **sp. n.**, and *L.schausi* Goldstein, **sp. n.** Images of adults and, where available, larvae as well as dissected genitalia are presented, with a key to adults.

## Introduction

*Chytonidia* Schaus, 1914 represents one of two noctuine genera (Lepidoptera: Noctuidae) described by Schaus (1914) that have recently been found feeding on ferns (Pteridophyta) as wild-caught caterpillars inventoried at Área de Conservación Guanacaste (ACG), northwestern Costa Rica. Fern-feeding (pteridivory) by herbivorous insects is of interest because the age and toxicity of ferns, coupled with the alleged rarity of fern specialists, have occasioned a modest literature that includes hypotheses of their origins (e.g., Balick et al. 1978, Cooper-Driver 1980, Hendrix 1980, Radhika et al. 2012). However, caterpillar sampling efforts during the last four decades in ACG (Janzen and Hallwachs 2016) have documented numerous pteridivorous caterpillars, most of them specialists and most previously unknown. In an effort to characterize better the prevalence of fern-feeding behaviors, as well as to serve the larger aims of Neotropical noctuid systematics, certain basic revisionary work necessary to examine the origins and extent of caterpillar pteridivory more precisely has been undertaken. This paper is a contribution to the systematics of noctuine genera now known to comprise pteridivores, our immediate purpose being to clarify the boundaries of one such genus, describe new species discovered through efforts at ACG, associate undetermined specimens with valid names, and collate life history information to the extent possible.

In the course of this work, existing collection material is complemented with recently reared specimens accompanied by life history data and DNA barcode data, the familiar ~657bp sequence from within the cytochrome oxidase I mitochondrial gene. These data coupled with comparative morphological observations, particularly those of the male genitalic complex, enable the revised taxonomic circumscriptions and descriptions of otherwise cryptic species presented here, and supplemented by a companion piece on the related pteridivorous genus *Lophomyra* Schaus, 1911 (Goldstein et al. 2018, this issue).

## Materials and methods

Pinned specimens were examined with an incandescent light source. Genitalic preparations follow Clarke (1941) in part and Lafontaine (2004), but staining with chlorazol black and slide mounting in euparal. Dissections followed either an overnight room-temperature soak in supersaturated sodium hydroxide or a 15 minute heated soak, and were examined under dissecting microscopes prior to slide mounting. Photographs were made using the Microptics and Visionary Digital imaging systems and images manipulated with Adobe Photoshop (Adobe Systems, Mountain View, CA). Measurements were made with the aid of an ocular micrometer. Forewing length was measured from the center of the axillary area to the apex of the forewing. Terminology generally follows Lafontaine (1998, 2004).

The descriptions by Druce (1908), Dognin (1910), and Schaus (1911, 1914) were thorough, although devoted primarily to wing pattern. Rather that reproduce them, genitalia and other characters not treated in the original descriptions are emphasized, and diagnoses generated for each species. Full descriptions are presented for all new species.

The complex, sometimes exaggerated structures associated with the male clasping apparatus requires that the terminology be clarified surrounding at least four sets of homologous structures common to all species examined. These are enumerated in the re-description of the genus and figured and labeled accordingly in Figures 111–126 of select specimens as reference. Because of their unusual configuration, associating these structures unambiguously to corresponding features in the traditional system of Forbes is not straightforward. Forbes himself noted in his introduction to the male genitalia of noctuids that “Here the problem is not to find characters for separation, but to find enough resemblances to make possible the identification of parts” (Forbes 1954: 12). In the case of *Leucosigma*, the appearance of several variously sclerotized structures within the valve complicates the differentiation of clasper from cucullus, for example. Rather than introduce any new vocabularies, our descriptions are anchored in the least ambiguous terms possible, explaining sources of potential ambiguity and our decisions concerning their treatment as they arise.

Provisional (neighbor-joining) analyses of DNA barcode data informed the dissection efforts and were supplemented by partial sequencing of DNA extracted during the course of dissection of the type of *Leucosigmauncifera*. That extraction involved an overnight soak of the abdomen in proteinase K and sequestration of the lysate prior to soaking the abdomen in KOH per the normal dissection protocol.

For specimens recently reared or collected from ACG, the following label data precede Santa Rosa National Park (SRNP) identifier codes, and are not repeated as label data except for newly designated types: Voucher: D.H. Janzen & W. Hallwachs DB: http://janzen.sas.upenn.edu Area de Conservacion Guanacaste, COSTA RICA.

### Repository abbreviations

The following abbreviations refer to collections from which specimens form the basis of this study:

**NHMUK** The Natural History Museum, London, UK (formerly BMNH)

**USNM** National Museum of Natural History [formerly, United States National Museum], Washington, District of Columbia, USA

## Systematics

Schaus (1914) designated *Chytonidiachloristis* Schaus, 1914 the type species of his monobasic genus *Chytonidia*, having described *Chytonixchloe* Schaus, 1914 one page prior. Poole (1989: 251) transferred *Chytonixchloe* to *Chytonidia*, along with *Miseliaalbimixta* Schaus, 1911, *Chytonixcommixta* Schaus, 1914, and *Gonodesviridipicta* Dognin, 1910. Although the genus was no longer monobasic, its nomenclatural composition was substantially reshaped. While transferring *commixta* to *Lophomyra* Schaus, 1911 in a separate paper (Goldstein et al. 2018, this issue), we here synonymize *Chytonidia* with *Leucosigma* Druce, 1908) based on a range of morphological characters and in particular similarities in male genitalia. Similarities in adult morphology, wing pattern, larval behavior and a small portion of the mitochondrial genome of *Leucosigma* species to species of *Chytonidia*, including both type species, require that *Leucosigma* be recognized as the senior synonym of *Chytonidia*.

### Leucosigma

Taxon classificationAnimaliaLepidopteraNoctuidae

Druce, 1908

Chytonidia Schaus, 1914, syn. n.

#### Type species.

*Leucosigmauncifera* Druce, 1908, by monotypy.

#### Etymology.

*Leuccosigma* was presumably named in reference to the white U-shaped forewing stigma. *Chytonidia* likely derives from *χιτών*, a coat, tunic or garment worn under a tunic, possibly in reference to thoracic and abdominal tufts.

#### Diagnosis.

Species of *Leucosigma* are diagnosed most readily by the male genitalia, in particular the nature of the highly differentiated clasping architecture, the most striking feature of which is the heavily sclerotized pincer-like cucullus on each valve. In referring to this as a cucullus, we do not interpret it as homologous to the clasper sensu Forbes; although derived from the basal sclerite, it is distinct if not entirely free of the more reduced clasper proper, which appears embedded within the sacculus and free of the basal sclerite. The cucullus bears a swollen, often bulb-like apex with a single heavily sclerotized spine, and a setose usually finger-like dorsal process (swollen apically in *L.viridipicta*). These most heavily sclerotized parts of the cucullus may appear simple and pincer-like as in *L.uncifera*, *L.poolei*, and *L.solisae*; recurved or falcate as in *L.albimixta*; asymmetrically distended with sinuate edges as in *L.chloe*; or elongate as in *L.schausi*.

#### Description.

*Head*. Labial palpus upturned, densely scaled; second segment usually longer than combined length of the first and third. Eyes smooth or sparsely hairy. Antennae setose-ciliate; bifasciculate in males, scaled above. *Thorax*. Collar green towards the base, the remainder of the thoracic vestiture more uniformly brown, but scales actually pale at the base. *Wings*. With the exception of the type species *L.uncifera*, which exhibits dominantly reddish or russet forewing coloration, the forewings of *Leucosigma* species are mottled with a mixture of brown and mossy green, particularly in the medial and terminal areas. Reniform and orbicular spots elongate and usually fused ventrally or nearly so, forming a roughly U-shaped stigma. At least four costal striae present. Sexual dimorphism in wing pattern is most conspicuous in the hind wings, which tend to be more uniformly dark gray in the females and paler basally in the males. *Legs*. Femoral and tibial scales always with an admixture of lime green among the tan and “lilacine” (cf. Schaus 1914: 488); a single pair of striped mid-tibial spurs, two pairs on hind-tibiae; three rows of tibial spines on all legs. *Abdomen*. Vestiture usually paler than on thorax and concolorous with hind wing surfaces. Abdomen without coremata, brushes, pockets or levers. *Male genitalia*. Uncus setose and variously tufted, crested, sinuate, or (in *L.viridipicta*) elongate and densely covered with clustered setae throughout. Tegumen hemi-circular to oblong, in some species raised at the base of the uncus. Vinculum shallow U- or V-shaped. Saccus bluntly pointed, rounded, or squared. Valves symmetrical, highly differentiated. For reference, we call attention to four structures numbered 1–4 in Figs 111–118 which account for much of the conspicuous diagnostic variation and which appear throughout the key and descriptions to follow: (1) the sacculus/saccular extension; (2) the cucullus, identified as such by virtue of reëntrant setae sensu Forbes (1954: 13) and what may be interpreted as an anal spine, as well as (3) a finger-like dorsal process which arcs dorso-medially *in situ* such that the apices of each process flank the uncus; and (4) a smaller finger-like, thorn-like or (in *L.viridipicta*) anvil-like clasper. Vesica unadorned, without spines or cornuti; a subbasal diverticulum ranges from rudimentary, as in *L.uncifera*, to a large recurved torsion in *L.albimixta*. *Female genitalia*. Papillae anales blunt, subquadrate. Posterior apophyses usually at least twice as long as anterior apophyses. Lamellae antevaginales robust, ranging from narrowly concave or cup-like to more deeply invaginate, apparently co-developed with colliculum. Colliculum usually well developed but reduced or absent in some species. Ductus bursae otherwise unsclerotized, straight or with minimal torsions, attaching dorso-caudally to the corpus, sometimes with a small pre-collicular lobe. Corpus bursae without signa, oblong, sometimes appearing subtriangular when distended by presence of spermatophore(s). Appendix bursae undifferentiated except as a swollen ventral point of attachment to the ductus seminalis. Spermatophore collum, when visible, appears with a single loop; frenum with a concave face matching the interior surface of appendix bursae at its point of attachment to the ductus seminalis. Paired pad-like sternal sclerotizations on A7 are visible in some specimens.

#### Distribution.

Mexico, Caribbean, Central and South America.

#### Biology.

All known caterpillars feed on leaves of ferns (Pteridophyta).

#### Remarks.

As is often the case, the type species of *Leucosigma* is perhaps the most phenotypically atypical of the genus. Known only from adult specimens taken at lights, the life history is unknown, although it may be reasonably suspected that the larva is a fern feeder. Species circumscription and nomenclatural assignment in this work has of necessity involved a combination of recently collected specimens for which DNA barcodes have been generated and historical specimens for which they have not, these latter including (for the present, at least) type specimens, several of which are females.

#### Key to known species of *Leucosigma* based on structures of the male genitalia.

Numbers 1–4 refer to structures labeled in figures.

**Table:** 

1	Saccular extension (1) with sclerotized point at apex; clasper (4) well developed, elongate, thorn-like or gently sinuate	*** L. schausi ***
–	Saccular extension not sclerotized apically; clasper (4) club-like, recurved and pointed, or anvil-like	**2**
2	Dorsal and ventral edges of cucullus (2) sinuate; clasper (4) recurved	*** L. chloe ***
–	Cucullus (2) modified distally to form simple hook- or chelicera-like point, either symmetrical or with deformation confined to the dorsal edge; clasper (4) recurved to a fine point, finger-like, or anvil-shaped	**3**
3	Distal end of cucullus aduncate; distance from vertex at outer edge to tip occupying less than 1/4 length of sclerotized part; clasper (4) simple, recurved, or otherwise modified	**4**
–	Distal end of cucullus (2) chelicerate, the outer edge of apex swollen such that distance to tip occupies ~1/3 length of heavily sclerotized part; clasper (4) rudimentary, finger-like, never sharply recurved or hooked	**6**
4	Distal part of cucullus (2) essentially straight without noteworthy angular deformation, ventral edge bulging; clasper (4) recurved and sharply pointed	*** L. reletiva ***
–	Distal part of cucullus (2) bent slightly backwards along outer edge, before hooking sharply inward at apex; clasper (4) not sharply hooked	5
5	Costal lobe (3) elongate, finger-like; costal process of the sacculus (4) simple	*** L. albimixta ***
–	Dorsal process (3) swollen at tip; cucullus (#2) with medially produced flange marking developmental separation from dorsal edge of clasper (4) which appears anvil-like	*** L. viridipicta ***
6	Inner edge of cucullus (2) distal to its fusion with dorsal process (3) straight for more than half its length before forming 90° angle/curve in its distal half	*** L. uncifera ***
–	Inner edge of cucullus (2) distal to its fusion with dorsal process (3) begins to curve in its basal half	**7**
7	Outer edge of cucullus (2) bending abruptly, the spine perpendicular to the long axis of the cucullar stem (cf. *L.uncifera*)	*** L. solisae ***
–	Outer edge of cucullus (2) bending gradually, the spine at an obtuse angle to the long axis of the cucullar stem	*** L. poolei ***

### Leucosigma uncifera

Taxon classificationAnimaliaLepidopteraNoctuidae

Druce, 1908

Figs 1–6 , 13 , 14 , 23 , 24 ; Male genitalia: Figs 53–56 , 77–80 , 111 , 112 ; Female genitalia: Fig. 105

Leucosigma uncifera Druce, 1908: 302. Type locality: Peru: [Puno] Carabaya, La Oroya.
Poole (1989) included Leucosigma Hampson, 1908 (preoccupied) as a junior synonym of Leucosigma Druce, 1908.

#### Material examined.

**Type material**. HOLOTYPE ♂: La Oroya, Carabaya, Peru, 3000 ft. iii. 1905. G. Ockenden., B. 433 4462, Brit. Mus. 1930-75, *Leucosigmauncifera* type, Druce, Type, NHMUK010606200. Type at NHMUK (BMNH).

#### Other material examined.

**Costa Rica**: (14♂,5♀): *Males*: Guanacaste (9♂): Sector Cacao: Cima, 10.93259, -85.45889, el. 1450m, 07/12/2010, F. Quesada & S. Rios, collector, 10-SRNP-111595, USNMENT01437310, 09-SRNP-100529, USNMENT01437201; Sector Pitilla: Estacion Pitilla, 10.98931, -85.42581, el. 675m, 03/03/2006, H. Cambronero & F. Quesada, collector, 06-SRNP-102855, USNMENT01437242; Sector Del Oro: Serrano, 11.00023, -85.45621, el. 585m, 11/08/2007, F. Quesada & S. Rios, collector, 07-SRNP-109808, USNMENT01437187; Sector Pitilla: Estacion Pitilla, 10.98931, -85.42581, el. 675m, 02/16/2007, F. Quesada & S. Rios, collector, 07-SRNP-100616, USNMENT01437320; Sector Pitilla: Estacion Pitilla, 10.98931, -85.42581, el. 675m, 02/17/2007, S Rios & F. Quesada, collector, 07-SRNP-101239, USNMENT01437200; Sector Pitilla: Estacion Pitilla, 10.98931, -85.42581, el. 675m, 02/16/2007, F. Quesada & S. Rios, collector, 07-SRNP-100629, USNMENT01438859; Sector Pitilla: Estacion Pitilla, 10.98931, -85.42581, el. 675m, 03/01/2006, S. Rios & R. Franco, collector, 06-SRNP-102374, USNMENT01438843, USNM Dissection 148199; Sector Cacao: Cima, 10.93259, -85.45889, el. 1450m, 07/12/2010, F. Quesada & S. Rios, collector, 10-SRNP-111594, USNMENT01437221; Sector Pitilla: Estacion Pitilla, 10.98931, -85.42581, el. 675m, 02/17/2007, S Rios & F. Quesada, collector, 07-SRNP-101266, USNMENT01437266; Alajuela (5♂): Sector Rincon Rain Forest: Rio Francia, 10.90425, -85.28651, el. 410m, 01/24/2009, R. Franco & S. Rios, collector, 09-SRNP-100529; Sector Rincon Rain Forest: Albergue Oscar, Casa, 10.86623, -85.32693, el. 719m, 01/03/2011, H. Cambronero & F. Quesada, collector, 11-SRNP-100029, USNMENT01438838, USNM Dissection 148102; Sector Rincon Rain Forest: Wege Palmeras, 10.96869, -85.31965, el. 369m, 10/22/2014, S. Rios & H. Cambronero, collector,14-SRNP-104353, USNMENT01437217; Sector Rincon Rain Forest: Albergue Oscar, Casa, 10.86627, -85.32605, el. 725m, 02/11/2010, S. Rios & F. Quesada, collector, 10-SRNP-104917, USNMENT01437285; Sector San Cristobal: Estacion San Gerardo, 10.88009, -85.38887, el. 575m, 10/11/2007, F. Quesada & R. Franco, collector, 07-SRNP-109451, USNMENT01370293, USNM Dissection 148101. *Females*: Costa Rica. Tuis. 2,500 ft. June. W. Schaus. 1910-110.*Leucosigmauncifera* Druce, NHMUK010606201; Guanacaste (4♀): Sector Pitilla: Estacion Pitilla, 10.98931, -85.42581, el. 675m, 02/28/2006, S. Rios & H. Cambronero, collector, 06-SRNP-101607, USNMENT01437350; Sector Pailas: Manta Copelares, 10.81692, -85.34679, el. 1478m, 09/07/2010, S. Rios & R. Franco, collector, 10-SRNP-113265, USNMENT01437197, USNM Dissection 148103; Sector Pitilla: Estacion Pitilla, 10.98931, -85.42581, el. 675m, 04/03/2011, F. Quesada & S. Rios, collector, 11-SRNP-102284, USNMENT01437255, USNM Dissection 148104; Sector Pitilla: Estacion Pitilla, 10.98931, -85.42581, el. 675m, 11/13/2012, S. Rios & H. Cambronero, collector, 12-SRNP-105657, USNMENT01370289.

#### Diagnosis.

The predominantly orange-russet forewing coloration and U-shaped solid white stigma diagnose this species from all others currently described; apical patch reduced to a white line extending from R5 to costa; post-medial line complete on underside of forewing, where terminal area appears dusted in green and contrasts with medial area; postmedial line almost complete on underside of hind wing. Cucullus pincer-like, the inner edge distal to its juncture with dorsal process straight for more than half its length before bending sharply and terminating in a sharp point.

#### Re-description.

***Head.*** Male and female antennae setose-ciliate with dorsal cupreous scales; male antennae bifasciculate. Frons, vertex, and basal two segments of labial palpus with orange/russet-colored scales; 3^rd^ segment palpus and antennal scape edged with white scaling. Eyes smooth.

***Thorax.*** Patagial “fans” appearing banded, two patches of green scales towards base, purplish medially, becoming paler or sunset-orange towards the crest; remainder of prothoracic vestiture a mix of purplish and russet scales. *Wings*. Forewing length 10.5 mm (holotype, male), average 10.3 mm (males, *n* = 4), 11.0 mm (females, *n* = 2). Predominantly russet orange; grayish purple in the spaces between R4 and R5, M1 and M3, and CuA2 and 1A. U-shaped stigma uniformly white. Moss-green scaling in the outer part of the apical patch and in the medial area towards the inner margin. Basal, antemedial, and postmedial lines white along inner edges, black outward; medial line more faint. Underside pattern elements more distinct than in other *Leucosigma* species): both forewing and hind wing postmedial lines visible, the postmedial area suffused throughout with green scaling. *Legs.* Scales predominantly pinkish; femoral and tibial scales with an admixture of lime green; a single pair of striped mid-tibial spurs, two pairs on hind-tibiae; three rows of tibial spines on all legs.

***Abdomen.*** Male dorsum covered uniformly in tannish-gray scales and hairs, predominantly lilac gray in females; abdominal underside more variably scaled with lilacine and reddish brown.

***Male genitalia.*** Uncus elongate, widest subapically, upwardly curved with a very small apical point, and bearing ventral setal crest. Tegumen raised at base of uncus. Vinculum a shallow V-shape; saccus bluntly pointed. Juxta pentagonal, dorsal edge horizontal; annellar arms fused, hoop-like. Sacculus (1) rounded apically, without sclerotized tip or point, its edges and those of dorsal process (3) setose; cucullus (2) appearing chelicerate, the sclerotized part occupying >1/3 its overall length but bending sharply at the outer edge near the sharply pointed apex with a subapical tuft of reëntrant spine-like setae; finger-like dorsal process (3) coequal in width to cucullus, to which it is joined midway; clasper (4) an elongate, bent free sclerite, embedded in sacculus. Distal part of aedeagus and basal part of vesica minimally spinulose, with small sclerotized apical flange and small, bilobate subbasal diverticulum.

***Female genitalia.*** Posterior apophyses less than twice as long as anterior apophyses. Lamella antevaginalis invaginated posteriorly, convex anteriorly. Colliculum undeveloped. Ductus undifferentiated, dimensionally intermediate for genus (cf. *L.chloe*, below), similar in length to 8^th^ abdominal segment. Corpus bursae elongate, obliquely bent, banana-shaped or sub-triangular when distended with spermatophore(s).

***Immature stages.*** Unknown.

#### Etymology.

Likely refers to the u-shaped forewing stigma.

#### Biology.

The life history of this species is unknown; to our knowledge, this species has never been reared, and has only been taken at lights. All SRNP-coded specimens with DNA barcodes examined were light-trapped in ACG rain forest.

#### Distribution.

Costa Rica, Peru. Specimens identified as *L.uncifera* are reported and have been DNA barcoded from Ecuador, Venezuela, and French Guiana.

#### Remarks.

The brightly colored orange-russet *Leucosigmauncifera*, the type species of *Leucosigma*, is the most visually distinct and atypical of the entire group, but the male genitalia are strikingly similar to those of *L.solisae*. DNA barcode data suggest the existence of undescribed species in a complex with *L.uncifera*, and it is not unlikely that additional sampling will reveal that the Costa Rican species are not conspecific with typical *L.uncifera*.

### Leucosigma reletiva

Taxon classificationAnimaliaLepidopteraNoctuidae

Dyar, 1914

Figs 19 , 20 , 29 , 30 ; Male genitalia: Figs 61 , 62 , 91 , 92 , 115 , 116 ; Female genitalia: Fig. 110

Leucosigma reletiva Dyar, 1914. Type locality: [Panama] Trinidad River, female.

#### Material examined.

**Type material**. HOLOTYPE ♀: **PANAMA**: A Busck coll, Rio Trinidad Mar. 12 Pan., Type No. 15839 U.S.N.M., *Leucosigmareletiva* Type Dyar, USNMENT00973166, ♀USNM Dissection 148170. Type at USNM.

#### Other material examined.

**COSTA RICA** (2♂): Alajuela: Sector Rincon Rain Forest: Protrero Chaves, 10.93868, -85.32167, el. 433m, 08/18/2009, R. Franco& S. Rios, collector, 09-SRNP-107116, USNM Dissection 148177, USNMENT01370297; Guanacaste: Sector Pitilla: Estacion Pitilla, 10.98931, -85.42581, el. 675m, 02/17/2007, S Rios & F. Quesada, collector, 07-SRNP-101206, USNM Dissection 148178, USNMENT01437211.

#### Diagnosis.

Wing patterning not readily differentiated from that of *L.chloe*, although overall paler in the specimens examined and figured here, possibly reflecting wear. Both *L.reletiva* and *L.chloe* have in common with *L.schausi* a reniform spot with a straight outer edge, squared at the lower corner. Male genitalia similar in some respects to those of *L.chloe*, especially in the recurved, pointed clasper, but sacculus wider and cucullus less sinuate apically, aduncate, with pronounced subapical setal fan as in *L.albimixta*; vesica with small distal secondary lobe.

#### Re-description.

***Head.*** Antennae setose-ciliate, bifasciculate in males, scaled above with alternating tan and brown bands of spatulate scales. Frons, vertex, and labial palpus with mixture of gray, brown, and some purplish scales; frons paler than vertex. Eyes sparsely hairy.

***Thorax.*** Thoracic vestiture a mix of grayish-tan scales tipped with brown, a pair of green-scaled patches at base of pagatial fan. *Wings.* Forewing length 11.8 mm (holotype, female), average 12.0 mm (males, *n* = 2). Forewing pale, slightly cupreous baso-medially such that the gray medial line stands out; fused reniform-orbicular stigmata green, outlined in white, bracket a brown patch outlined in dark brown; terminal area shaded with green; apical patch silvery white; hind wings uniformly gray above in male and female alike, hind wing undersides shaded towards costa, postmedial line broken. *Legs.* Scales grayish brown, mostly concolorous with thoracic vestiture; femora and tibia with an admixture of lime-green scales; a single pair of striped mid-tibial spurs, two pairs on hind-tibiae; three rows of tibial spines on all legs, as for other members of the genus.

***Abdomen.*** Dorsum covered in uniformly tannish-brown scales and hairs; ventral side more darkly scaled with two rows of paler tan scales on either side of the medial line (cf. *L.chloe*, below).

***Male genitalia.*** Uncus elongate, widest subapically, upwardly curved with a small apical point. and bearing ventral crest of short setae. Tegumen hemi-circular or nearly so. Vinculum cup-shaped. Saccus squared off. Juxta spade-shaped, with a mid-dorsal projection; annellar arms fused, hoop-like. Sacculus (1) bluntly rounded, heavily setose; sclerotized part of cucullus (2) elongate, concave, aduncate with a subapical setal fan; dorsal process (3) widest medially, densely setose apically; clasper (4) recurved to a point. Aedeagus faintly granular towards vesica, where the sclerotized part is narrowed to a sinuous strap. Vesica without cornuti; paired subbasal and medial diverticula knoblike, with a small apical diverticulum present.

***Female genitalia.*** Posterior apophyses less than twice as long as anterior apophyses. Lamella antevaginalis V-shaped, deeply invaginated posteriorly. Ostium wide. Colliculum welldeveloped. Ductus undifferentiated except for small pre-collicular lobe. Corpus bursae obliquely shaped, sub-triangular when distended with spermatophore(s). Ductus bursae robust, not >3× long as width at middle. Colliculum present, well developed. With two spermatophores, maintaining a very roughly foot-shaped appearance, the distal (anterior) part of the bursa distended to accommodate the corpus of the spermatophore (cf. holotype of *L.uncifera*).

***Immature stages.*** Undocumented.

#### Biology.

Feeding on foliage of *Bolbitisportoricensis* (Dryopteridaceae) in ACG rain forest (96-SRNP-11467).

#### Distribution.

Costa Rica, Panama.

#### Remarks.

Dyar described this species on the basis of a single female holotype specimen in poor condition which he nevertheless recognized as having kinship with *L.uncifera*.

### Leucosigma albimixta

Taxon classificationAnimaliaLepidopteraNoctuidae

(Schaus, 1911) comb. n.

Figs 39 , 40 , 49 , 50 ; Male genitalia: Figs 66–68 , 85–87 ; Female genitalia: Fig. 106 ; Larvae: Figs 131–134

Chytonidia albimixta Schaus, 1911 Type locality: Costa Rica.

#### Material examined.

**Type material**. Holotype ♀: *Miselia* sp. not in USNM, *Miseliaalbimixta* type Schs, Type No. 17326 [526?] USNM, Juan Vinas CR, May, USNM Dissection 148184, USNMENT01370283. Type at USNM.

#### Other material examined

(5♂, 1♀). **COSTA RICA** (4♂): Alajuela (2♂): Sector Rincon Rain Forest: Albergue Oscar, Casa, 10.86627, -85.32605, el. 725m, 01/15/2010, F. Quesada & S. Rios, collector, 10-SRNP-104564, USNM Dissection 148,305, USNMENT01370298; Sector Rincon Rain Forest: Manta Hugo, 10.8811, -85.2677, el. 491m, 03/15/2009, H. Cambronero & F. Quesada, collector, 10-RNSP-107587, USNM Dissection 148069, USNMENT01437230. Guanacaste (2♂): Sector Pitilla: Estacion Pitilla, 10.98931, -85.42581, el. 675m, larva on *Elaphoglossumdoanense*: 02/14/2011, ecl. 03/29/2011, Manuel Rios, collector, 11-SRNP-30511, USNM Dissection 148085, USNM 00105321; Sector Pitilla: Estacion Pitilla, 10.98931, -85.42581, el. 675m, 03/18/2007, H. Cambronero & S. Rios, collector, 07-SRNP-102199, USNM Dissection 148068, USNMENT01437365 **GUATEMALA** (1♂, 1♀): Chejel Guat, Aug, Schaus and Barnes coll., ♂ genitalia on slide Aug 1953 E.L.T. #138, ♂ USNM Dissection 50,548, USNMENT01437345; Chejel Guat, Aug, Schaus and Barnes coll., ♀ genitalia on slide Aug 1953 E.L.T. #139, ♀ USNM Dissection 50,549, USNMENT01370282.

#### Diagnosis.

Largest *Leucosigma* species, forewing approaching 1.5cm on average (see below), and the only species with most of the medial area of the forewing upperside dominated by green; the terminal area also with green scaling, the components of the fused reniform-orbicular swollen and outlined in white, appearing more bulbous than in any other species except the much smaller *L.viridipicta*, with which *L.albimixta* is not likely confused; terminal area well defined on hind wing underside, more extensively shaded in green than in other species; clasper swollen, fan-like and concave medially with short acutely recurved apical hook. Ductus bursae narrow as in *L.poolei* but point of attachment to corpus bursae dorsad; colliculum diminutive as in *L.uncifera* and *L.poolei*.

#### Re-description.

***Head.*** Antennae setose-ciliate, bifasciculate in males, scaled above with alternating tan and brown bands of spatulate scales. Scaling on frons, vertex, and labial palpi predominantly brown, interspersed with black and white. Labial palpus with third segment relatively longer than in congeners, almost half the length of the second segment. Eyes sparsely hairy, with a post-ocular ring of dark-purplish hair-like scales.

***Thorax.*** Collar green scaled; a sublabial green beard-like tuft observed in one male specimen (USNM 00105321). *Wings*. Forewing length 14.9 mm (holotype, female), average 14.4 mm (males, *n* = 5), 14.8 mm (females, *n* = 2). Medial and terminal areas of forewing dominated by green, especially so the distended “U” comprising the fused reniform-orbicular complex; basal and postmedial areas dominated by brownish-gray scaling; underside patterning concentrated in costal and terminal areas, with neither a discal spot nor robust antemedial or postmedial lines apparent excepting a partial postmedial on the forewing. *Legs.* Green scale tufts on inner mid- and hind-femora, the remaining femoral scales white, brown, and black, and the tibial scaling primarily green and white; a single pair of striped mid-tibial spurs, two pairs on hind-tibiae; three rows of tibial spines on all legs.

***Abdomen.*** Uniform grayish tan above; underside a mix of white and light/dark brown with medial patches of green.

***Male genitalia.*** Uncus elongate, upwardly curved with a small apical point and bearing ventral setal crest. Tegumen ∩-shaped. Vinculum widely V-shapped. Saccus squared off. Juxta dihedral, subquadrate with the dorsal and ventral edges deformed in parallel (the dorsal invaginated and the ventral evaginated); annellar arms fused, hoop-like. Sacculus (1) simple, tapered; cucullus (2) curved outward apically before recurving to a fine point, supapical setal fan prominent; dorsal process (3) elongate, gently tapered, setose throughout; clasper (4) upturned, apex foot-shaped. Aedeagus with a sclerotized band of raised granules and a more distal patch of spinules. Vesica without cornuti; subbasal diverticulum bulbous; medial diverticulum an enlarged simple torsion.

***Female genitalia.*** Posterior apophyses nearly twice as long as anterior apophyses. Lamella antevaginalis deeply invaginated posteriorly. Ostium narrow. Colliculum reduced. Ductus elongate, narrow, >0.5× length of corpus bursae, itself elongate, obliquely shaped; appendix bursae bulbous.

***Immature stages.*** Known only from images (Figs 131–134). The larva of *L.albimixta* is predominantly light green with a fine, pale ramifying pattern and a series of raised, rust-colored lateral spiracular patches; a broken white supra-spiracular line encloses the antero-ventral half of a small dark spot above each spiracle; dorsal pinacula raised; head capsule yellow with scattered red markings; dorsum of metathoracic segment tapers downward from anterior part of A1 at 45° angle.

#### Biology.

**Figures 85–92.**
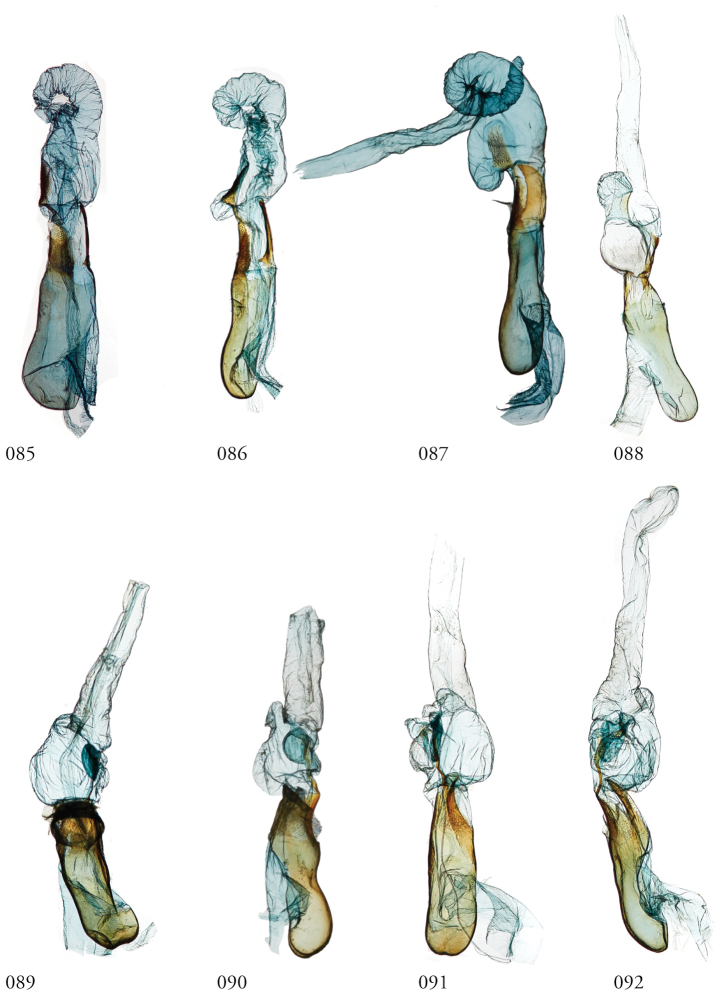
*Leucosigma* male genitalia, phalli. **85***L.albimixta*, Área de Conservación Guanacaste (ACG), Costa Rica,*a* 11-SRNP-30511, USNM Dissection 148085, USNM 00105321 **86***L.albimixta* 10-RNSP-107587, USNM Dissection 148069,USNMENT01437230 **87***L.albimixta*USNM Dissection 148305, USNMENT01370298_ **88***L.viridipicta* Holotype, French Guiana, USNM Dissection 148176, USNMENT 00973419 **89***L.chloe*, ACG, 96-SRNP-11375, USNM Dissection 148070, USNMENT01438814 **90***L.chloe*, ACG, 96-SRNP-11369, USNM Dissection 148289, USNMENT01437372_ **91***L.reletiva*, ACG, 09-SRNP-107116, USNM Dissection 148177, USNMENT01370297 **92***L.reletiva*USNM Dissection 148178, USNMENT01437211.

**Figures 111–114.**
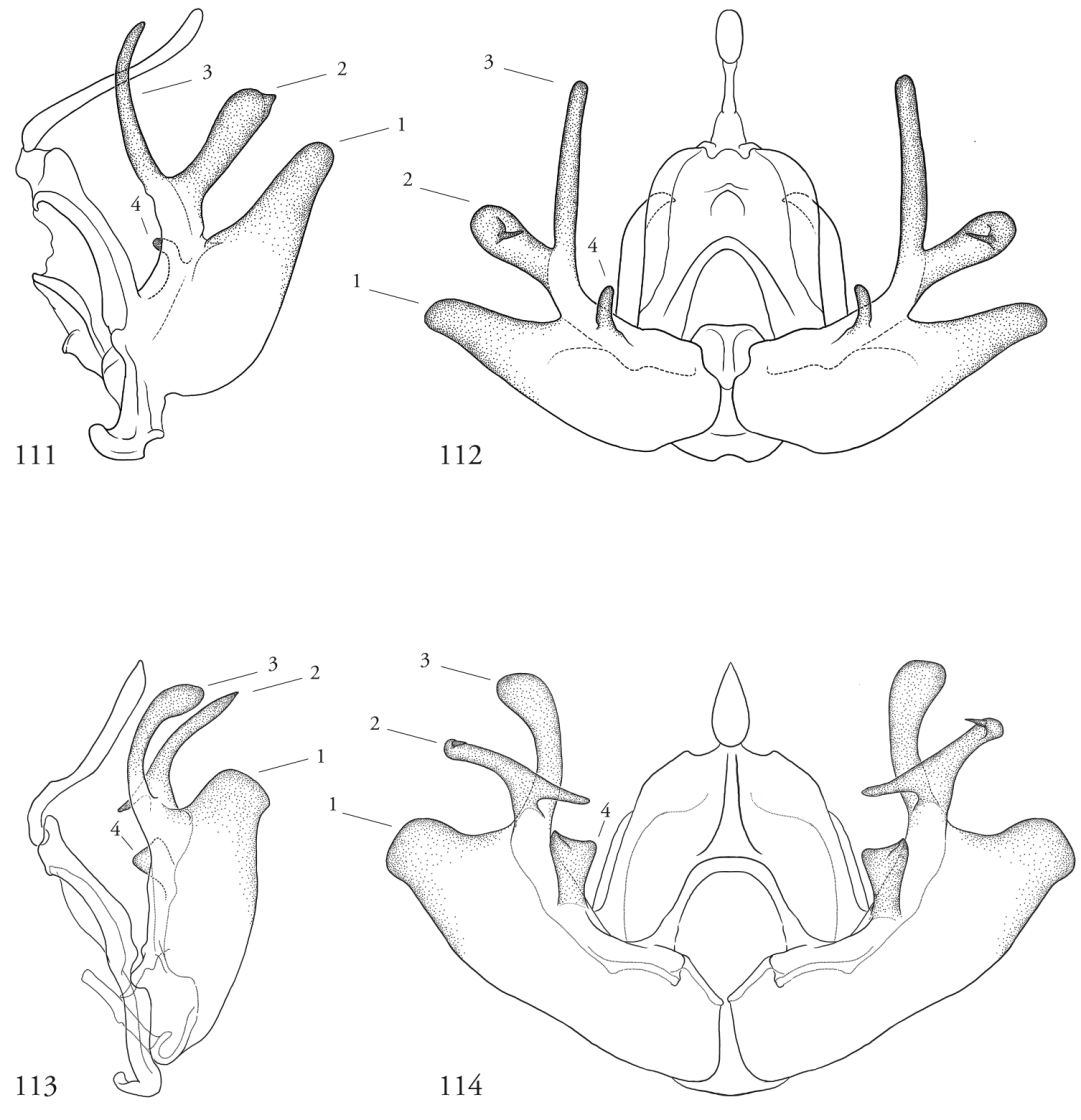
Representative male valvae, structures enumerated as in text: (1) sacculus (2) cucullus (3) dorsal process of cucullus and (4) clasper. **111–112***L.uncifera* ♂ Holotype, Área de Conservación Guanacaste (ACG), Costa Rica, 07-SRNP-109451, USNMENT01370293, ♂ USNM Dissection 148101 **111** Lateral **112***C*audal **113***L.viridipicta* Holotype ♂, USNMENT 00973419,USNM Dissection 148176, Lateral **114***L.viridipicta* Holotype ♂, USNMENT 00973419, USNM Dissection 148176, Caudal.

**Figures 115–118.**
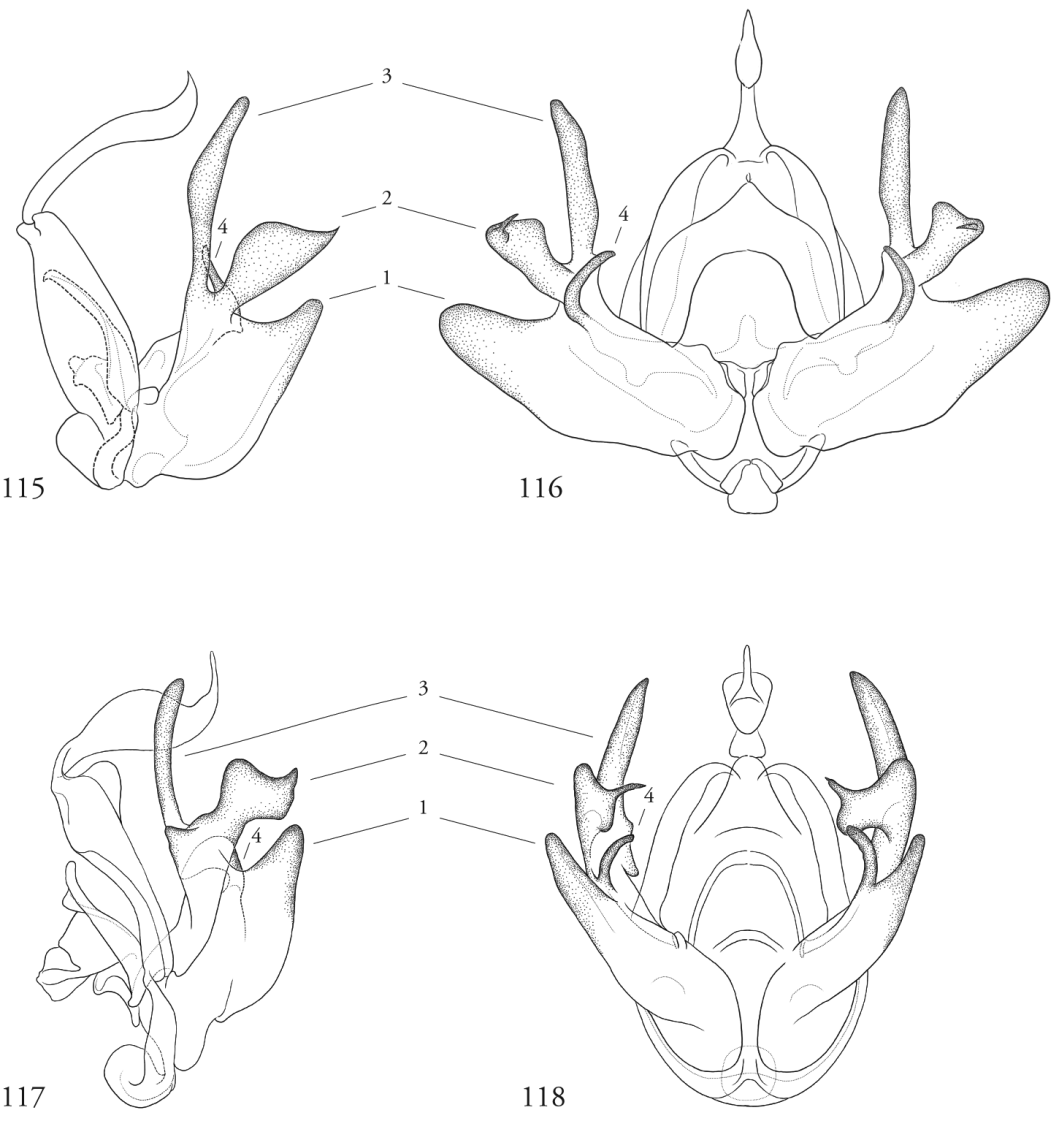
Representative male valvae, structures enumerated as in text: (1) sacculus (2) cucullus (3) dorsal process of cucullus and (4) clasper. **115–116***L.reletiva* ♂, Área de Conservación Guanacaste (ACG), Costa Rica, 07-SRNP-101206, USNM Dissection 148178, USNMENT01437211 **115** Lateral **116** Caudal **117–118***L.chloe*, ACG, 10-SRNP-70815, USNMENT01437250, USNM Dissection 148200 **117** Lateral **118** Caudal.

**Figures 119–123.**
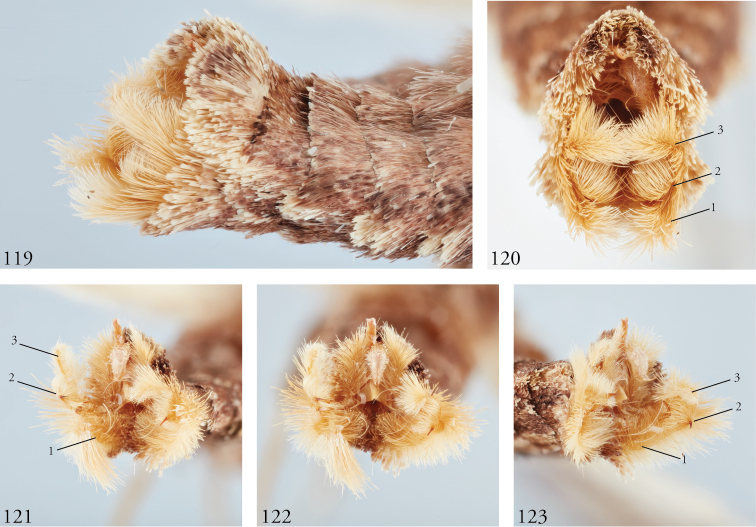
*Leucosigmachloe* male terminalia illustrating orientation of tufts *in situ*, with structures labeled as in previous figures to indicate (1) sacculus; (2) cucullus and (3) dorsal process. **119–120***L.chloe*, Área de Conservación Guanacaste (ACG), Costa Rica,96-SRNP-11373, USNMENT01437360 **119** Lateral **120** Caudal **121–123***L.chloe*, ACG, 96-SRNP-11371, USNMENT01370286.

Caterpillar found feeding on foliage of *Elaphoglossumdoanense* (Dryopteridaceae) in ACG rain forest. In the reared male specimen for which data are available (11-SRNP-30511; Figs 66, 85, 131–134), 26 days elapsed between the prepupal stage and adult eclosion. Other ACG specimens were light-trapped.

#### Distribution.

Costa Rica, Guatemala.

### Leucosigma chloe

Taxon classificationAnimaliaLepidopteraNoctuidae

(Schaus, 1914) comb. n.

Figs 7–12 , 21 , 22 , 31 , 32 ; Male genitalia: Figs 63 , 64 , 89 , 90 , 117 , 118 (terminalia in situ), 119–123 ; Female genitalia: Figs 100 , 101 , 107 , 129 , 130 (abdominal terminus, ventral aspect)

Chytonix chloe Schaus, 1914. Type locality: French Guyana.
Chytonidia chloristis Schaus, 1914: 489.

#### Material examined.

***Type material.*** HOLOTYPE ♀: St Jean, Maroni, Fr Guiana, *Chytonixchloe* Type Schs, Type No. 16531 U.S.N.M., Collection Wm Schaus, USNM Dissection 148175, USNMENT01370295. Type at USNM.

#### Other material examined.

(11♂, 14♀). **FRENCH GUIANA** (2♀):[HOLOTYPE of *Chytonidiachloristis*, syn. of *chloe*]: ♀ St Jean, Maroni, Fr Guiana, *Chytonidiachloristis* Type Schs, Collection Wm Schaus, Type No. 16533 U.S.N.M., USNM Dissection 148185, USNMENT01370300; Dates 6-8 Mar 1982 Guyane Francaise G. Tavasillian [illeg.] leg. BORWE I [illeg.], 376, USNMENT01370375.

**COSTA RICA** (11♂, 11♀): *Males*: COSTA RICA: Turrialba 17-21.II.65 SS&WD Duckworth, USNM Dissection 148151, USNMENT01437357 [♂]; Guanacaste (10♂): Sector Cacao: Estacion Gongora, 10.887, -85.47443, el. 570m, larva on *Bolbitisportoricensis*: 11/10/1996, ecl. 10/21/1996, 96-SRNP-11370, USNMENT01437307; Sector Cacao: Estacion Gongora, 10.887, -85.47443, el. 570m, larva on *Bolbitisportoricensis*: 11/12/1996, ecl. 10/21/1996, 96-SRNP-11369, USNM Dissection 148,289, USNMENT01437372; Sector Pitilla: Estacion Quica, 10.99697, -85.39666, el. 470m, larva on *Microgrammapercussa*: 03/18/2010, ecl. 02/15/2010, Ricardo Calero, collector, 10-SRNP-70814, USNMENT01437206; Sector Pitilla: Estacion Quica, 10.99697, -85.39666, el. 470m, larva on *Microgrammapercussa*: 03/21/2010, ecl. 02/15/2010, Dinia Martinez, collector, 10-SRNP-70815, USNM Dissection 148200, USNMENT01437250; Sector Cacao: Estacion Gongora, 10.887, -85.47443, el. 570m, larva on *Bolbitisportoricensis*: 11/13/1996, ecl. 10/21/1996, 96-SRNP-11375, USNM Dissection 148070, USNMENT01438814; Sector Cacao: Estacion Gongora, 10.887, -85.47443, el. 570m, larva on *Bolbitisportoricensis*: 11/12/1996, ecl. 10/21/1996, 96-SRNP-11372, USNM Dissection 148071,USNMENT01437315; Sector Pitilla: Estacion Quica, 10.99697, -85.39666, el. 470m, larva on *Microgrammapercussa*: 08/22/2010, ecl. 07/23/2010, Ricardo Calero, collector, 10-SRNP-72329, USNM Dissection 148168, USNMENT01437257; Sector Cacao: Estacion Gongora, 10.88449, -85.47306, el. 557m, 09/11/2007, R. Franco & S. Rios, collector, 07-SRNP-108955, USNM Dissection 148055, USNMENT01437367; Sector Cacao: Estacion Gongora, 10.887, -85.47443, el. 570m, larva on *Bolbitisportoricensis*: 11/11/1996, ecl. 10/21/1996, 96-SRNP-11373, USNMENT01437360; Sector Cacao: Estacion Gongora, 10.887, -85.47443, el. 570m, larva on *Bolbitisportoricensis*: 11/13/1996, ecl. 10/21/1996, 96-SRNP-11371, USNMENT01370286.

*Females*: Guanacaste (9♀): Sector Cacao: Estacion Gongora, 10.887, -85.47443, el. 570m, larva on *Bolbitisportoricensis*: 10/21/1996, ecl. 11/21/1996, 96-SRNP-11376, USNMENT01437271; Sector Pitilla: Estacion Quica, 10.99697, -85.39666, el. 470m, larva on *Microgrammapercussa*: 07/20/2010, ecl. 08/17/2010, Ricardo Calero, collector, 10-SRNP-72238, USNMENT01437347; Sector Pitilla: Estacion Quica, 10.99697, -85.39666, el. 470m, larva on *Microgrammapercussa*: 07/22/2010, ecl. 08/19/2010, Dinia Martinez, collector, 10-SRNP-72308, USNMENT01437272; Sector Pitilla: Quebradona, 10.99102, -85.39539, el. 475m, larva on *Microgrammapercussa*: 02/04/2010, ecl. 03/10/2010, Ricardo Calero, collector, 10-SRNP-70655, USNMENT01437302; Sector Pitilla: Estacion Quica, 10.99697, -85.39666, el. 470m, larva on *Microgrammapercussa*: 08/02/2010, ecl. 09/11/2010, Ricardo Calero, collector, 10-SRNP-72520, USNMENT01437280; Sector Pitilla: Estacion Quica, 10.99697, -85.39666, el. 470m, larva on *Microgrammapercussa*: 11/07/2010, ecl. 12/20/2010, Ricardo Calero, collector, 10-SRNP-73250, USNMENT01437292; Sector Pitilla: Estacion Quica, 10.99697, -85.39666, el. 470m, larva on *Microgrammapercussa*: 07/13/2010, ecl. 08/15/2010, Ricardo Calero, collector, 10-SRNP-72038, USNM Dissection 148072, USNMENT01437382; Sector Pitilla: Estacion Quica, 10.99697, -85.39666, el. 470m, larva on *Microgrammapercussa*: 02/04/2012, ecl. 03/07/2012, Ricardo Calero, collector, 12-SRNP-70275, USNM Dissection 148054, USNMENT01437297; Sector Pitilla: Estacion Quica, 10.99697, -85.39666, el. 470m, larva on *Microgrammapercussa*: 07/16/2013, ecl. 08/10/2013, Ricardo Calero, collector, 13-SRNP-71183, USNMENT01437287. Alajuela (2♀): Sector Rincon Rain Forest: Estacion Botarrama, Manta Porton, 10.96048, -85.28237, el. 147m, F. Quesada & R. Franco, collector, 09-SRNP-108544, USNM Dissection 148169, USNMENT01437380; Sector Cacao: Estacion Gongora, 10.887, -85.47443, el. 570m, larva on *Bolbitisportoricensis*: 10/21/1996, ecl. 11/12/1996, 96-SRNP-11374, USNMENT01370299.

**BRAZIL** (1♀): Sao Paulo de Olivenca Amazones Fassl Novembre-Decembre, USNM Dissection 148152, USNMENT01437322

#### Diagnosis.

Forewing similar to that of *L.reletiva* but forewing overall more darkly shaded in specimens examined; reniform spot with a straight outer edge, squared at the lower corner as in *L.reletiva* and *L.schausi*; male hind wing pale basally in males, not uniformly shaded as in *L.reletiva*. Cucullus with highly distinct asymmetrical deformations along both edges subapically, the apical point (≈ anal spine) ventrally curved.

#### Re-description.

***Head.*** Antennae setose-ciliate, bifasciculate in males, scaled above with indistinctly banded tan and brown scales. Frons, vertex, and labial palpi with scales heterochromously shaded in tannish brown. Eyes sparsely hairy.

***Thorax.*** Prothoracic vestiture brown, sprinkled with green; dorsal tuft well developed. *Wings.* Forewing length 11.8 mm (holotype, female), average 11.3 mm (males, *n* = 8), 11.7 mm (females, *n* = 8). *Legs.* Femora thickly scaled with brown and black, the mid- and hind-femora with green scaling in addition; tibiae uniformly less densely scaled, in pale tan; a single pair of striped mid-tibial spurs, two pairs on hind-tibiae; three rows of tibial spines on all legs.

***Abdomen.*** Dorsum covered in uniformly tannish-brown scales and hairs; dorso-lateral tufts of hairs on anterior segments; ventral side more darkly scaled with two rows of paler tan scales on either side of the medial (cf. *L.reletiva*, above).

***Male genitalia.*** Most similar to *L.uncifera* in size, but with the shape of the cucullus at its apex highly distorted, asymmetrical. Uncus crested, distended medially, upwardly turned with long apical point and ventral crest of short setae. Tegumen with dorsal edge nearly straight, the lateral edges outwardly sub-parallel. Vinculum cup-shaped. Saccus bluntly triangulate. Juxta spade-shaped, with a mid-dorsal projection; annellar arms fused, hoop-like. Sacculus (1) tapered to a dull point, the dorsal and ventral edges straight. Edges of cucullus (2) asymmetrical, sinuate, apical spine produced along ventral edge; dorsal process (3) undifferentiated. Clasper (4) robust, recurved to a point (cf. *L.reletiva*). Aedeagus with distal sclerotization faintly strap-like, and minimally rugose, somewhat granular towards vesica. Vesica likewise faintly granular basad, without cornuti; subbasal diverticulum moderately distended with a pair of smaller lobes and a pouch-like invagination with a weakly sclerotized margin as in *L.reletiva*.

***Female genitalia.*** Posterior apophyses more than twice as long as anterior apophyses. Lamella antevaginalis invaginate. Colliculum well developed. Ductus intermediate for genus, 2–4× as long as wide. Corpus bursae sub-triangular when distended; appendix bursae a small bulbous out-pouching.

***Immature stages.*** No images or specimens available.

#### Biology.

Caterpillars found in ACG rain forest feeding on foliage of *Bolbitisportoricensis* (Dryopteridaceae) and *Microgrammapercussa* (Polypodiaceae). Of 20 reared specimens (6 males, 14 females) an average of 19 days elapsed between the onset of the prepupal stage and adult eclosion, with most individuals requiring 19 or more days.

#### Distribution.

Costa Rica, French Guiana.

#### Remarks.

*Leucosigmachloe*, its synonym (and the type species) *L.chloristis*, and *L.reletiva* were all described from female holotypes.

### Leucosigma viridipicta

Taxon classificationAnimaliaLepidopteraNoctuidae

(Dognin, 1910) comb. n.

Figs 41 , 42 , 51 , 52 ; Male genitalia: Figs 65 , 88 , 113 , 114 ) 127 , 128

Gonodes viridipicta Dognin, 1910: 13. Type locality: French Guiana: St. Laurent du Maroni.

#### Material examined.

***Type material.* FRENCH GUIANA**: HOLOTYPE ♂; S. -Laurent de Maroni Guy Franc; Dognin collection, *Gonodesviridipicta* 1/10 Type ♂ Dognin not in USNM [illeg.], Type No. 32413 U.S.N.M., ♂USNM Dissection 148176, USNMENT00973419. Type at USNM.

#### Other material examined.

(1♂). **PERU**: Huacamayo, Carabaya, dry seas., 3100 ft, June 04. (G. Ockenden), Rothschild Bequest B.M. 1939-1, NHMUK01606202.

#### Diagnosis.

Smaller than *L.albimixta*, with fused orbicular and reniform spots similarly swollen, but with the basal, antemedial, and postmedial lines less conspicuously highlighted in black and white. Cucullus with medial flange directed basad, its ventral edge precisely complementing the dorsal edge of clasper from which it appears to have been separated during development; apices of the costal lobes swollen.

#### Re-description.

***Head.*** Antennae setose-ciliate, bifasciculate in males, scaling above uniformly grayish brown. Vertex and labial palpi with scales predominantly grayish brown; frons and inner face of palpus with paler scaling. Eyes smooth.

***Thorax.*** Prothoracic scales grayish brown, concolorous with vertex. *Wings.* Forewing length 10.9 mm (holotype, male), average 10.8 mm (males, *n* = 2). Apical patch dominantly green on forewing upperside and underside, and on underside of hind wing. *Legs*– Scaling predominantly tannish brown, more or less concolorous with thoracic vestiture; a single pair of striped mid-tibial spurs, two pairs on hind-tibiae; three rows of tibial spines on all legs.

***Abdomen.*** Dorsum covered in uniformly tannish-brown scales and hairs; ventral side more darkly scaled, especially at terminal tuft; some pinkish scaling ventrally.

***Male genitalia.*** Uncus robust and densely setose, almost bottlebrush-like for distal 3/4, the setae blonde, concolorous with neighboring setal tufts, and arranged in clusters sharing a single socket and shingled, appearing scale-like *in situ*. Tegumen raised at base of uncus. Vinculum laterally concave. Saccus blunt. Juxta rhomboid, without a dorsal projection. Sacculus (1) wide, barely tapered. Cucullus (2) bent backward (ventro-cephalad) and bears basally directed flange marking separation from dorsal edge of clasper, evidently ruptured during development of especially robust dorsal processes (3); each dorsal process swollen apically, resembling a ball-headed Native American war club, heavily setose, and with a conspicuous tuft of ventro-medially directed spine-like setae. Clasper (4) anvil shaped, its dorsal edge complementing the ventral edge of the cucullar flange. Aedeagus weakly sclerotized, granular appearance continuing to base of vesica; subbasal diverticulum asymmetrically bulbous, dumbbell-shaped.

***Female genitalia.*** Unknown.

***Immature stages.*** Unknown.

#### Biology.

Unknown.

#### Distribution.

French Guiana, Peru.

#### Remarks.

Female specimens at MNHN (Paris) were not available for study. This species is noteworthy in that the uncus is covered in shingled, scale-like clusters of setae (Figs 65, 127, 128), reminiscent of other fern-feeding species in the genus *Lophomyra*. The relationship between these genera requires more thorough sampling and analysis of both taxa and genes.

### Leucosigma solisae

Taxon classificationAnimaliaLepidopteraNoctuidae

Goldstein sp. n.

http://zoobank.org/C50ACC24-E816-4019-8FF5-7690574D9D3B

Figs 15 , 16 , 25 , 26 ; Male genitalia: Figs 57 , 58 , 81 , 82

#### Material examined.

***Type material.*** HOLOTYPE ♂. Voucher: D.H. Janzen & W. Hallwachs DB: http://janzen.sas.upenn.edu Area de Conservacion Guanacaste, COSTA RICA, Sector Pitilla: Estacion Pitilla, 10.98931, -85.42581, el. 675m, 02/17/2007, S. Rios & F. Quesada, collector, 07-SRNP-101229, ♂USNM Dissection 148077, USNMENT01370294.

PARATYPES (2♂). **COSTA RICA**: (1♂): Alajuela: Sector Rincon Rain Forest: Estacion Caribe, 10.90082, -85.2764, el. 391m, 10/10/2007, S. Rios & H. Cambronero, collector, 07-SRNP-109201, ♂USNM Dissection 148078, USNMENT01437370. **PERU** (1♂): La Oroya, R. Inambari, Peru, Sept. 1904 3100 ft, dry seas., (G. Ockenden), Rothschild Bequest B.M. 1939-1., NHMUK010606203. Types at USNM.

#### Diagnosis.

Forewing terminal area and distal part of the medial area appearing more uniform purplish gray than in other species, giving the wing a smoother, less granular appearance overall. The reniform-orbicular complex forms a continuous green “Ч “(left wing) or “μ” (right wing), the outline of which is broader than in other species except *L.albimixta* and *L.viridipicta*; the green (not silvery-white) apical patch distinguishes this and the following species from other members of the genus; hind wing underside with discal spot present but unpronounced, unlike *L.poolei* below. Male genitalia most similar to those of *L.poolei* and *L.uncifera*, distinguished by a shorter and more gently arced distal part of the cucullus; vesica without small basal lobe as in *L.poolei*.

#### Description.

***Head.*** Antennae setose-ciliate, bifasciculate in males, scaled above with alternating bands of gray (basal) and tan (apical). Frons, vertex, and labial palpi scaled with an admixture of white, brown and black. Eyes smooth.

***Thorax.*** Excepting vestiges of green visible immediately behind head, thoracic vestiture uniformly purplish gray. *Wings.* Forewing length 13.7 mm (holotype, male), average 13.3 mm (males, *n* = 3). Forewing predominantly lilacine, russet subcostally in the postmedial; apical and anal patch present; green scaling concentrated at inner margin, apical and anal patch, and Ч/μ-shaped medial stigma. Medial area cupreous; medial, antemedial, and postmedial lines visibly darker brown; terminal area green in Costa Rican specimens, more closely matching medial coloration or slightly darker reddish brown in Peruvian specimen; apical patch primarily green; forewing underside lightly suffused with green scaling in terminal area; no antemedial or postmedial lines present on underside except at most as costal striae. *Legs.* Scales predominantly purplish gray; femora and tibia with an admixture of lime-green among the tan-lilacine scales; a single pair of striped mid-tibial spurs, two pairs on hind-tibiae; three rows of tibial spines on all legs.

***Abdomen.*** Vestiture uniformly tannish gray, paler than on thorax.

***Male genitalia.*** Uncus elongate, widest subapically, upwardly curved with a very small apical point, and bearing ventral setal crest. Tegumen roughly hemi-circular, excepting a deformation of the dorsal edge at the base of the uncus. Vinculum a wide V-shape; saccus blunt. Juxta pentagonal, dorsal edge horizontal; annellar arms fused, hoop-like. Sacculus (1) densely setose, tapering to a blunt extension. Cucullus (2) appears chelicerate, the sclerotized part occupying ~1/3 the overall length and bending sharply at the outer edge near the sharply pointed apex, with a subapical tuft of reëntrant spine-like setae. Dorsal process (3) coequal in width to cucullus, setose apically. Clasper (4) finger-like, gently curved. Aedeagus with minutely but differentially spinulose patch confined to apex. Vesica without cornuti; subbasal and medial diverticula reduced, with a weakly sclerotized ridge.

***Female genitalia.*** Unknown.

***Immature stages.*** Unknown.

#### Etymology.

The name *solisae* is given in honor of Dr. Alma Solis, lepidopterist at USDA/USNM who has contributed her expertise to the systematics of the Costa Rican lepidopteran fauna for three decades.

#### Biology.

Unknown, collected only in rain forest light traps.

#### Distribution.

Costa Rica, Peru.

### Leucosigma poolei

Taxon classificationAnimaliaLepidopteraNoctuidae

Goldstein sp. n.

http://zoobank.org/13EEBE89-A3B2-4B05-93E9-B8181A9B1CC7

Figs 17 , 18 , 27 , 28 ; Male genitalia: Figs 59 , 60 , 83 , 84 ; Female genitalia: Figs 102 , 103 ; Larvae: Figs 147–154

#### Material examined.

***Type material.*** (6♂, 2♀). **COSTA RICA**: HOLOTYPE: ♀ Voucher: D.H. Janzen & W. Hallwachs DB: http://janzen.sas.upenn.edu Area de Conservacion Guanacaste, COSTA RICA, Sector Pitilla: Quebradona, 10.99102, -85.39539, el. 475m, larva on *Microgrammapercussa*: 02/09/2010, Ricardo Calero, collector, 10-SRNP-70737, ♀ USNM Dissection 148073, USNMENT01370296.

PARATYPES (5♂, 1♀): Sector Pitilla: Estacion Quica, 10.99697, -85.39666, el. 470m, larva on *Microgrammapercussa*: 10/02/2010, ecl. 11/10/2010, Ricardo Calero, collector, 10-SRNP-73038, ♀ USNM Dissection148074, USNMENT01438839; Ibid, ♂ [abdomen missing]; Sector Pitilla: Sendero Rotulo, 11.01355, -85.42406, el. 510m, larva on *Elaphoglossumdoanense*: 07/10/2010, ecl. 08/17/2010, Manuel Rios, collector, 10-SRNP-31675, USNMENT01438823; COSTA RICA: Turrialba 22-28.II.65 SS & WD Duckworth, ♂USNM Dissection 148147, USNMENT01370290; Ibid, ♂USNM Dissection 148149, USNMENT01437186; Ibid, 1-6.III.65, ♂USNM Dissection 148150, USNMENT01437330; Ibid, ♂USNM Dissection 148148,USNMENT01438829. Types at USNM.

#### Diagnosis.

Forewing comparable in size but slightly larger on average than that of *L.chloe*, smaller than *L.solisae*; upperside pattern intermediate between the two in several respects, sharing the Ч/μ-shaped stigma of *L.solisae* rather than the straight/squared reniform of *L.reletiva* and *L.chloe*, but with the stigma narrow as in those latter species and not as swollen as in *L.solisae*. Likewise the overall appearance is more granular than *L.solisae* but with less conspicuous black edging or black wedges at the postmedial line than in *L.chloe* or *L.reletiva*. Hind wing underside with pronounced discal spot, ringed in black in both sexes. *Male genitalia*: Cucullus terminates in a rounded point, less acutely curved apically than in *L.uncifera* or *L.solisae*; inner edge distal to its fusion with finger-like dorsal process curving more gradually beginning in its basal half, the part distal to its articulation with the dorsal lobe the shortest among these three species.

#### Description.

***Head.*** Antennae setose-ciliate, bifasciculate in males, scaled above with alternating bands of gray (basal) and tan (apical). Frons, vertex and labial palpi scaled with an admixture of white, brown and black, tipped with paler scaling. Eyes smooth.

***Thorax.*** Thoracic vestiture chocolate brown. *Wings.* Forewing length 10.9 mm (holotype, male), average 11.4 mm (males, *n* = 4), 11.0 mm (females, *n* = 2). Forewing dominated by chocolate-brown coloration. Hind wing underside with pronounced discal spot ringed in black in both sexes. *Legs.* Scaling predominantly grayish brown or purplish gray with an admixture of green especially on the hind femora; a single pair of striped mid-tibial spurs, two pairs on hind-tibiae; three rows of tibial spines on all legs.

***Abdomen.*** Vestiture uniformly tannish gray, paler than on thorax.

***Male genitalia.*** Similar overall both to *L.uncifera* and *L.solisae*. Uncus elongate, widest subapically, upwardly curved with a very small apical point, and bearing ventral setal crest. Tegumen dome-shaped, except its dorsal edge deformed at the base of the uncus. Vinculum a wide V-shape; saccus bluntly rounded. Juxta pentagonal, dorsal edge horizontal; annellar arms fused, hoop-like. Sacculus (1) densely setose, tapered; saccular extension without sclerotized point. Cucullus (2) appears chelicerate, the sclerotized part occupying ~1/3 the overall length, widest medially and arcing gently before tapering to a sharply pointed apex with a subapical tuft of reëntrant spinelike setae. Dorsal process (3) coequal in width to cucullus, setose apically. Clasper (4) finger-like, gently curved. Uncus with ventral setal crest along distal half. Vesica with small basal secondary lobe. Aedeagus with an elongate sclerotized band of raised granules. Vesica without cornuti; paired and medial subbasal diverticular lobes nipple-like.

***Female genitalia.*** Posterior apophyses less than twice as long as anterior apophyses. Lamella antevaginalis invaginated. Colliculum undeveloped. Ductus elongate, narrow as in *L.albimixta*. Corpus bursae oblong.

***Immature stages.*** Known only from images (Figs 147–154). Young caterpillars sparsely setose, particularly on dorsal pinacula; A1 with a medial white spot flanked by a pair of yellowish or cream-colored subdorsal spots that are echoed on A8 but all of which vanish in later instars; mature larva rusty brown above to the lateral line, mottled green below; dorsal markings a series of medial blackish triangles narrowed caudally, each narrow end “cupped” in a lightly mottled mossy green; the effect is one of a series of v-shaped wedges, tapering caudally and enclosed at the narrow/caudal end with green mottling; diffuse brown subdorsal coloration darkens laterally to form an undulating line with each lighter brown abdominal “trough” enclosing a spiracle; the wavy black line separates the brown spiracular triangles from their complementary, dorsally directed green counterparts, continuous with the green venter and expanding with the increasingly distended posterior segments; head capsule brown with two black facial stripes on either side of the frons; antennae yellow.

#### Etymology.

The name *poolei* is given in gratitude to Dr. Robert Poole, noctuidologist and former curator at USNM, who first identified this and other species from among the reared Costa Rican material.

#### Biology.

Caterpillars found feeding on foliage of *Elaphoglossumdoanense* (Dryopteridaceae) and *Microgrammapercussa* (Polypodiaceae). Two reared male and female specimens (10-SRNP-31675 and 10-SRNP-73038) required 22 and 30 days, respectively, from the onset of the pre-pupal stage to adult eclosion.

#### Distribution.

Costa Rican rain forest.

#### Remarks.

Although the female genitalia are distinctive in the configuration of the ductus, the three recently reared specimens of *L.poolei*, comprising two females and a male with a dissociated abdomen, appear conspecific with four male specimens collected by S.S. and W.D. Duckworth in 1965, and cluster closely with both *Leucosigmasolisae*, known only from two males, and the two specimens referred to *L.reletiva*.

### Leucosigma schausi

Taxon classificationAnimaliaLepidopteraNoctuidae

Goldstein sp. n.

http://zoobank.org/B2CBD402-81BE-41F7-A55B-FF0046F2DBC9

Figs 35 , 36 , 37 , 38 , 45 , 46 , 47 , 48 ; Male genitalia: Figs 69–72 , 96 , 97 ; Female genitalia: Figs 104 , 108 ; Larvae: Figs 139–146.

#### Material examined.

***Type material.* COSTA RICA** (3♂, 3♀): HOLOTYPE ♂. Voucher: D.H. Janzen & W. Hallwachs DB: http://janzen.sas.upenn.edu Area de Conservacion Guanacaste. Guanacaste: Sector Pitilla: Estacion Quica, 10.99697, -85.39666, el. 470m, larva on *Microgrammapercussa*: 07/19/2010, ecl. 08/16/2010, Ricardo Calero, collector, 10-SRNP-72230, ♂USNM Dissection 148076, USNMENT01370303 PARATYPES (2♂, 3♀): *Males*: Sector Pitilla: Calma, 11.00987, -85.39214, el. 412m, 02/11/2010, ecl. 03/12/2010, Ricardo Calero, collector, 10-SRNP-70740, USNMENT01437325, USNM Dissection 148298; Sector Pitilla: Quebradona, 10.99102, -85.39539, el. 475m, larva on *Microgrammapercussa*: 02/04/2010, Ricardo Calero, collector, 10-SRNP-70653, USNMENT01437290 [abd. missing] *Females*: Sector Pitilla: Estacion Quica, 10.99697, -85.39666, el. 470m, larva on *Microgrammapercussa*: 06/23/2013, ecl. 07/17/2013, Ricardo Calero, collector, 13-SRNP-71023, USNM Dissection 148080, USNMENT01437332; Sector Pitilla: Quebradona, 10.99102, -85.39539, el. 475m, larva on *Microgrammapercussa*: 01/07/2010, ecl. 02/18/2010, Calixto Moraga, collector, 10-SRNP-70113, USNMENT01370292; Sector Pitilla: Estacion Quica, 10.99697, -85.39666, el. 470m, larva on *Microgrammapercussa*: 06/26/2010, ecl. 07/31/2010, Ricardo Calero, collector, 10-SRNP-71934, USNMENT01437312. Types at USNM.

#### Other material examined

(3♂, 7♀). **COSTA RICA** (1♂): Golfito 25-28.IV.65 SS & WDD DUCKWORTH, ♂USNM Dissection 148146, USNMENT01370291 **GUATEMALA** (2♂, 3♀): *Males*: Cayuga Guat, June, Schaus and Barnes coll, June, USNM Dissection 41,194, USNMENT01437362; Ibid, April, USNM Dissection 4091, USNMENT01437241 *Females*: Cayuga, Guat., July, Schaus and Barnes coll, *Chytonidiachloristis* Schs WT 6.21, USNMENT01437337; GUATEMALA Dept. Suchitepequez Cutotenango 10-20 June 1966 Flint &B Ortiz, USNMENT01437317; Cayuga, Guat, Aug, Schaus and Barness coll, USNMENT01437377 **CUBA** (2♀): Baracoa, Cuba, Collection Wm Schaus, ♀ genitalia Slide USNM Noc 4092, USNMENT01370281; Ibid, Oct., ♀ genitalia Slide USNM Noc 4093, USNMENT01437340 **MEXICO** (1♀): MEX: Tmps Gomez Farias 21 III 1981, Nacimiento del Rio Frio, Gillespy & Lara Collectors, USNMENT01437352 **PANAMA** (1♀): Bocas dToro Pan, Apr ‘07, Collection Wm Schaus, USNMENT01437300

#### Diagnosis.

Most readily differentiated from other *Leucosigma* by the sharply pointed sclerotized saccular extentions and the elongate cucullus reminiscent of the mandibles of trap-jaw ants (*Odontomachus* Latreille). Forewing similar to those of *L.chloe* and *L.reletiva*, the similarities including the pale apical patch, more variably shaded with green in *L.schausi*, and the straight/squared reniform, appearing more “pinched” into two trianguloid green wedges in *L.schausi*. Terminal area of hind wing underside distinctly paler than basal and medial areas but less extensively scaled with green than *L.albimixta*.

#### Description.

***Head.*** Antennae setose-ciliate, bifasciculate in males, finely scaled above with pinkish tan. Frons, vertex and labial palpi with variously hued brown and tan scales; scaling of the palpi paler underneath and on apical segment. Palps with brown and dark brown scales, paler on apical segment. Eyes sparsely hairy.

***Thorax.*** Anterior part of tegulae green. *Wings.* Forewing length 12.2 mm (holotype, male), average 12.2 mm (males, *n* = 8), 12.8 mm (females, *n* = 5). Pattern elements visible and distinct; variation in hue of brown shading a function of scale density, as the scales are more darkly colored at their tips; U-shaped fusion of reniform-orbicular complex similar to that in *L.poolei*, above; costal striae at basal, antemedial, and postmedial lines consist of juxtaposed black and white, white distal to black in the basal and postmedial striae and black distal to white in the antemedial stria; outermost costal striae white; postmedial line a series of black crescents enclosing pale brown centers; terminal line a series of more compressed such crescents; apical patch predominantly white with some green shading; underside shading concentrated in costal and especially terminal areas of both wings; lines incomplete, typical of genus. *Legs.* Most thickly scaled on femora, with mixture of colors similar to those of head predominantly brown and pinkish tan; femoral and tibial scales with an admixture of lime green; a single pair of striped mid-tibial spurs, two pairs on hind-tibiae; three rows of tibial spines on all legs.

***Abdomen.*** Vestiture uniform pale gray, more or less concolorous with hind wings; slightly darker ventrally.

***Male genitalia.*** Uncus elongate, widest subapically, upwardly curved with a conspicuous apical point, and bearing ventral crest of short setae. Tegumen dome-shaped, with its dorsal edge deformed at the base of the uncus. Vinculum shallow; saccus bluntly rounded. Juxta pentagonal, dorsal edge horizontal; annellar arms fused, hoop-like. Sacculus (1) tapering to a heavily sclerotized point; costal lobe of sacculus densely setose. Cucullus (2) elongate, straight for much of its length but curving apically to a point, with an expanded subapical setal patch. Adjoining dorsal process (3) setose, undifferentiated, coequal in width to cucullus. Clasper (4) elongate, thorn-like or gently sinuate, rendering the valvae with three pair of sharply pointed structures. Aedeagus with an elongate sclerotized band of raised granules. Vesica without cornuti; a small patch of granular spinules evident basad; subbasal diverticulum recurved, appearing trilobate, with medial diverticular “nipple” and a weakly sclerotized C-shaped ridge.

***Female genitalia.*** Posterior apophyses less than twice as long as anterior apophyses. Lamella antevaginalis invaginate. Colliculum well developed. Ductus robust, opening to corpus bursae wide. Corpus bursae subtriangulate when distended.

***Immature stages.*** Known from images of reared specimens (Figs 139–146). Larva predominantly light brown, faintly shaded with green intersegmentally and laterally on A4–6; coloration generally more uniform above spiracular line, reticulate below; the familiar dorsal herring-bone pattern consisting of a row of Y-shaped markings, the arms of which terminate at the D1 pinacula, which on A1 are partially encircled with anterior cream-colored marking to form a spectacle-like mark. Thoracic segments taper dorsally. Young larvae predominantly greenish gray, with anastomosing pattern visible dorsally; thin white spiracular line beneath a broader reddish-purple supraspiracular line, the upper edge of which runs just above SD1 pinacula.

#### Etymology.

The name *schausi* honors William Schaus, Jr. (January 11, 1858 – June 20, 1942), USDA entomologist and curator at USNM who described both *Chytonidia*, herein synonymized with *Leucosigma*, and the genus *Lophomyra*, also associated with ferns.

#### Biology.

Caterpillars found feeding on foliage of rain forest *Microgrammapercussa* (Polypodiaceae) in ACG. Six reared specimens required an average of 22.5 days between the onset of the prepupal stage to adult eclosion.

#### Distribution.

Costa Rica, Cuba, Guatemala, Mexico, Panama.

##### Other specimens of *Leucosigma*

(4♂, 1♀). Figs 33, 34, 43, 44; Male genitalia: Figs 73, 74, 93, 124–126 (*terminalia in situ*); Female genitalia: Fig. 109. Larvae: Figs 135–138.

**Figures 124–126.**
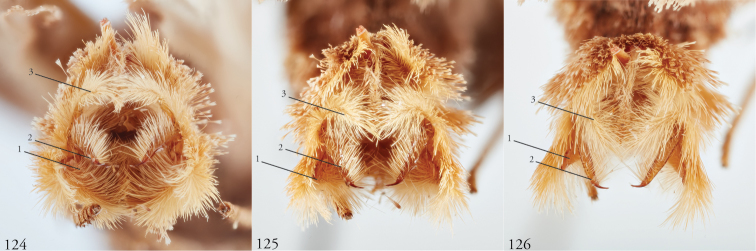
Leucosigmasp. nearschausi, terminalia *in situ*, caudal view from a progressively dorsal angle. Structures labeled as in previous figures to indicate (1) sacculus; (2) cucullus and (3) dorsal process. DOMINICAN REPUBLIC: Dajabon Province 13km S. Doma de Cabrera ca. 400 m, 20–22 May 1973 Don & Mignon Davis.

**Figures 127–128.**
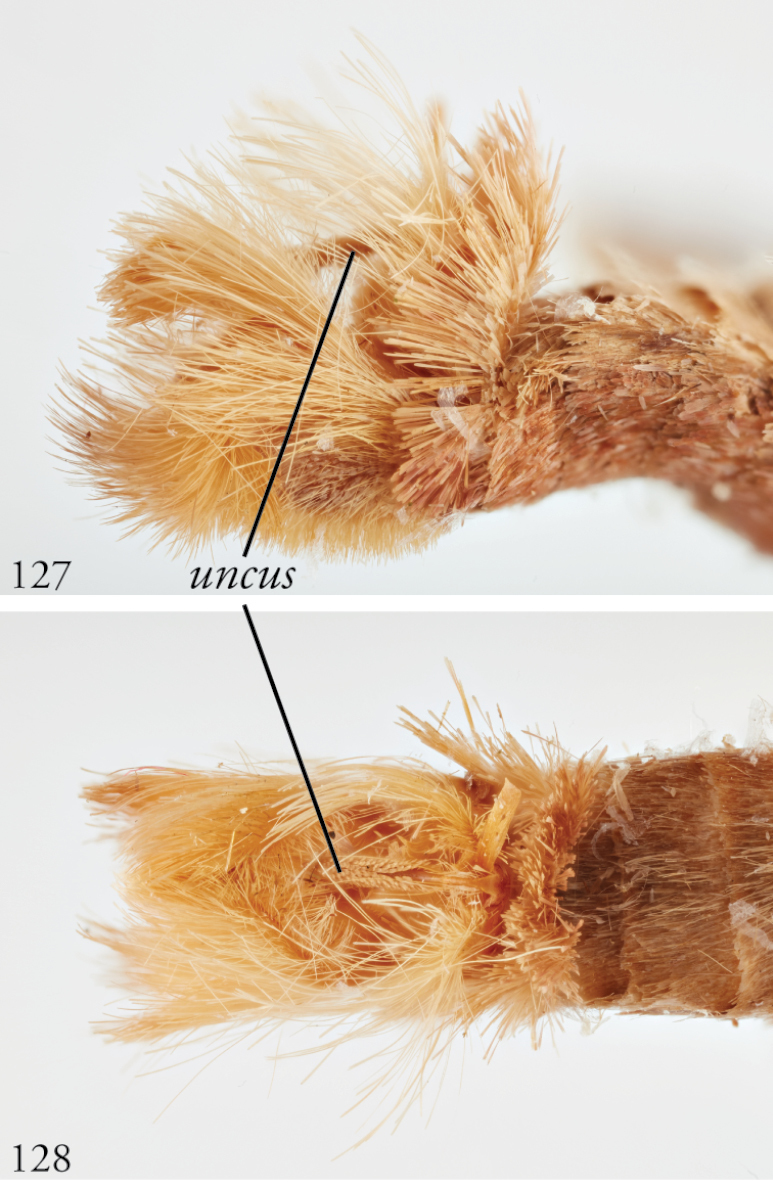
*Leucosigmaviridipicta* Holotype terminalia *in situ*, indicating clustered setae on the uncus. **127** Lateral **128** Dorsal.

**Figures 131–138.**
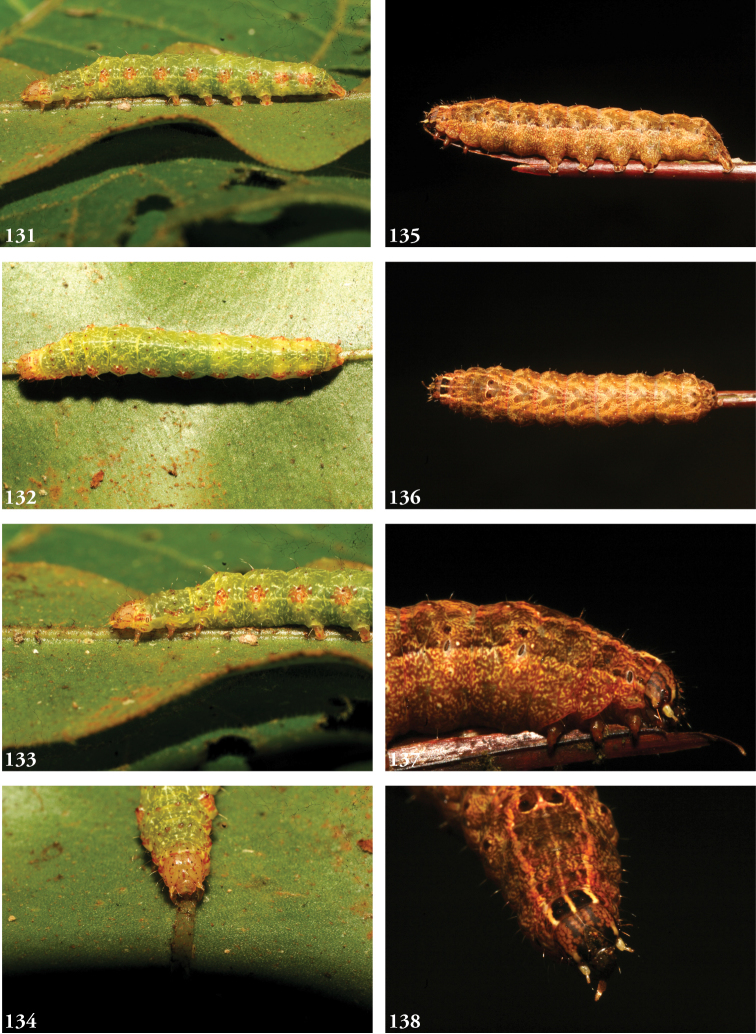
*Leucosigma* larvae, Área de Conservación Guanacaste (ACG), Costa Rica. **131–134***L.albimixta* ♂ 11-SRNP-30511 cf. Figs 66, 83**1131** DHJ481612 **132** DHJ48162 **133** DHJ481613 **134** DHJ481621 **135–138**Leucosigmasp. nearschausi, ♀11-SRNP-44977 cf. Figs 34, 44, 104**135** DHJ489159 **136** DHJ489157 **137** DHJ489154 **138** DHJ489160.

**Figures 139–146.**
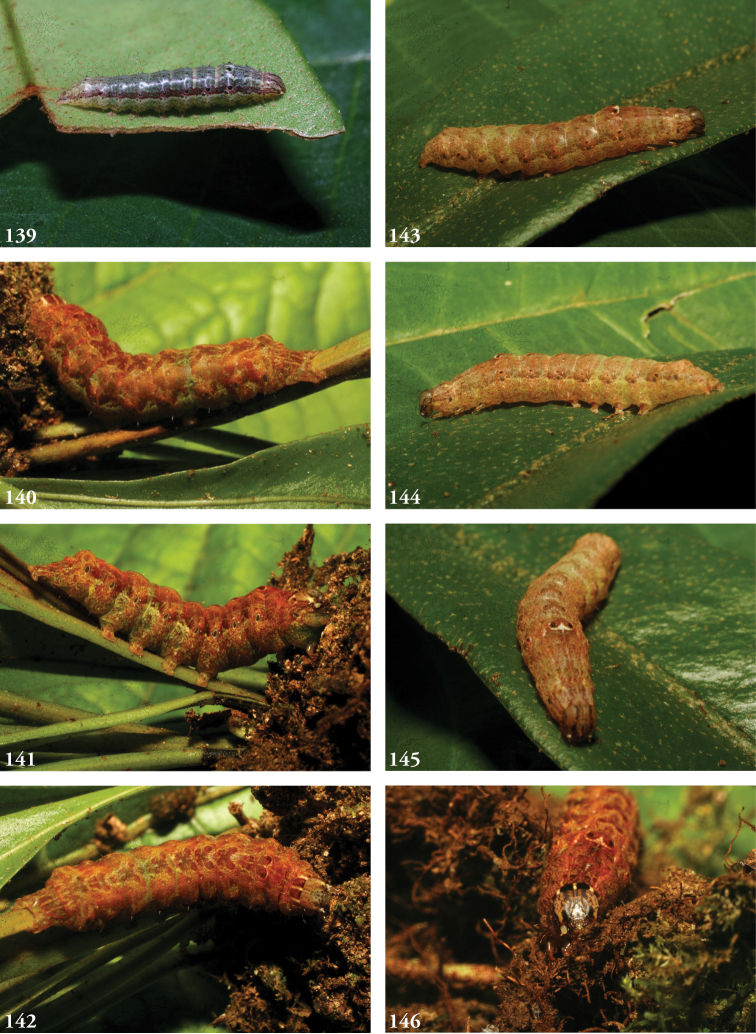
*Leucosigmaschausi*, larvae, Área de Conservación Guanacaste (ACG), Costa Rica. **139** ♂10-SRNP-70653-DHJ469071 **140–142** ♂ 10-SRNP-70740 **140** DHJ469099 **141** DHJ469108 **142** DHJ469109 **143–146** ♀ 10-SRNP-70113, USNMENT01370292 cf. Figs 36, 46**143** DHJ467595 **144** DHJ467593 **145** DHJ467592 **146** DHJ467604.

**Figures 147–154.**
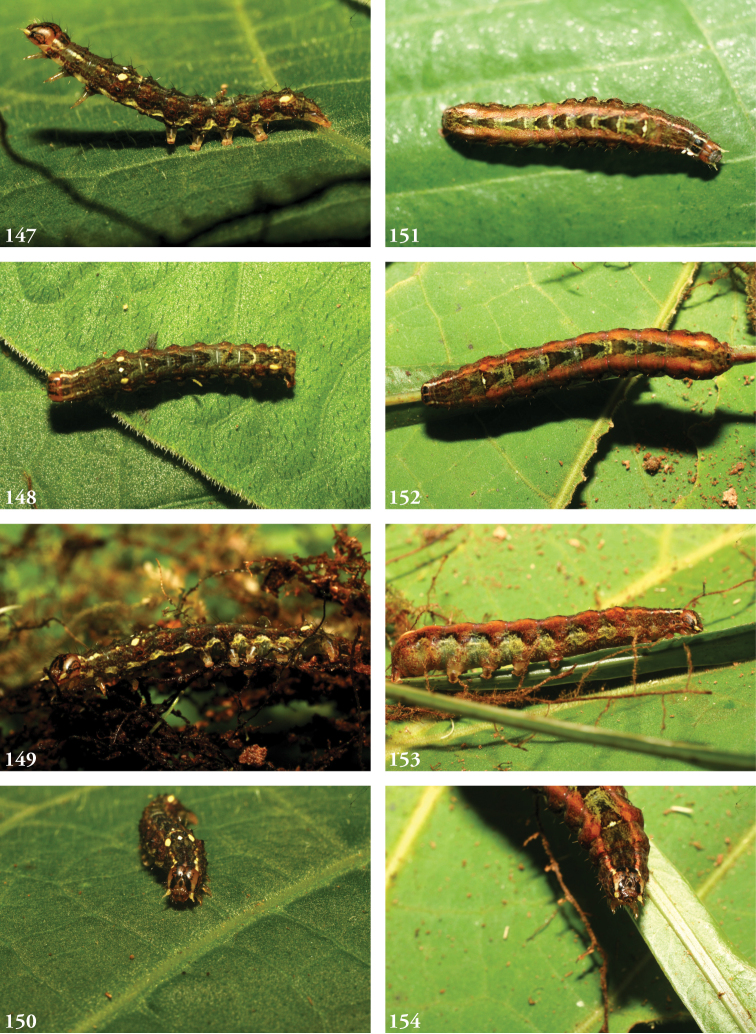
*Leucosigmapoolei*, larvae, Área de Conservación Guanacaste (ACG), Costa Rica. **147–150, 152–154** ♂10-SRNP-31675 **147** DHJ473617 **148** DHJ4 7361 **149** DHJ473609 **150** DHJ473620 **151** ♀10-SRNP-70737-DHJ469091 cf. Figs 18, 28, 97**152** DHJ47362 **153** DHJ473626 **154** DHJ47363.

Several specimens from the Dominican Republic bear similarities to a subset of those reared from ACG; all may represent variation within *L.schausi*, but because they bear minor genitalic differences consistent with barcodes that differ minimally (a single base pair) from those of *L.schausi*, the possibility that they represent a cryptic species remains open. The male genitalia differ slightly from those of *L.schausi* in the reduced sclerotized point at the apex of saccular extension. If in fact they prove to be distinct, they would be similar to the species pair *Neoxeniadesluda* and *Neoxeniadespluviasilva* (Hesperiidae), which co-occur at ACG and differ by only one base pair in their DNA barcode and very slight genitalic differences (Burns et al. 2007). It was decided not to describe a new species based on these characters, but rather to figure specimens and summarize information associated with them as “sp. near *L.schausi*”: **DOMINICAN REPUBLIC** (2♂): ♂, Dajabon Province 13km S. Roma de Cabrera ca. 400m, 20–22 May 1973 Don & Mignon Davis, ♂ USNM Dissection 148142, USNMENT01370285; ♂ Ibid, ♂ USNM Dissection 148297, USNMENT01437231, Male genitalia imaged *in situ* (Figs 124–126). **COSTA RICA** (2♂, 1♀): [♂] Guanacaste: Sector Pitilla: Estacion Quica, 10.99697, -85.39666, el. 470m, larva on *Microgrammapercussa*: 11/03/2010, ecl. 12/12/2010, Ricardo Calero, collector, 10-SRNP-73224, USNMENT0105325; [♂] Alajuela: Sector Rincon Rain Forest: Jacobo, 10.94076, -85.3177, el. 461m, larva on *Microgrammapercussa*: 01/17/2011, ecl. 02/14/2011, Edwin Apu, collector, 11-SRNP-69183, USNM Dissection 148075, USNMENT01438819; [♀] Alajuela: Sector Rincon Rain Forest: Estacion Caribe, 10.90187, -85.27495, el. 415m, larva on *Campyloneurumlatum*, 12/04/2011, Jorge Hernandez, collector, 11-SRNP-44977, USNM Dissection 148079, USNMENT01370288 7224.

## Discussion

The synonymy of *Chytonidia* with *Leucosigma* is straightforward; the circumscription of species boundaries perhaps less so. The predominance of female types among species in the *L.chloe* complex (*L.chloe*, *L.chloristis*, *L.reletiva*) is unfortunate given the predominance of conspicuous diagnostic features in males. The identification of modern Costa Rican material as conspecific with *L.reletiva* was made in the absence of historical specimens of males and of barcode data from the female holotype; if that determination is incorrect, then *L.reletiva* is likely another synonym of *L.chloe* and the male Costa Rican specimens will represent an undescribed species. Likewise, the pairing of an exclusively female sample of *L.poolei* with an exclusively male series of museum specimens is justified primarily on the basis of wing pattern, and our selection of a female holotype in this case draws from the fact that the specimen is accompanied both by barcode and rearing data.

No doubt that several species of *Leucosigma* remain undescribed, probably including at least one within the *L.uncifera* complex. Provisional (neighbor-joining) analyses of DNA barcode data corroborated the unity of *Leucosigma* and *Chytonidia* by virtue of the proximity of Costa Rican *L.uncifera* to species of *Chytonidia*, as did a partial sequence of the holotype of *L.uncifera* when analyzed against a sample of 1,355 noctuid specimens that inlcuded all the available known fern feeders. Since no single mitochondrial marker would be considered an adequate foundation for phylogenetic inference regardless of the analysis, we interpret analyses of COI data with caution (particularly beyond the level of closely related species), but with that said, we also note that the available barcode data are consistent with the hypothesis of *Leucosigma* monophyly delineated here.

Among the more intriguing morphological features are those surrounding the male clasping architecture, specifically the complex of variously sclerotized lobes and the associated arrangement of setal tufts. The possible function of these tufts as courtship structures bears further study, as do the mechanics of the unusually elaborate clasping appendages themselves. The as yet undetermined phylogenetic proximity of *Leucosigma* to the smaller of Schaus’ pteridivorous genera *Lophomyra* bears on our interpretation of the collective diet breadths of each, and to whether structures such as the clustered setae on the uncus in *Leucosigmaviridipicta* represents a symplesiomorphous condition homologous to that in *Lophomyra*.

In comparison with other noctuid genera now known to comprise fern-feeding species at ACG and elsewhere in the Neotropics, the phylogenetic or taxonomic breadth of host plants recorded for *Leucosigma* is quite narrow, restricted to Polypodiales and specifically the families Polypodiaceae and Dryopteridaceae. These represent the two most widely recorded families among Neotropical pteridivorous noctuid genera at ACG, most of which are known from a much broader range of fern families. The effort to document hostplant associations of Neotropical caterpillars supplements our understanding of unusual genera and of the distribution of fern-feeding. Although less biologically rare than previously supposed, pteridivory appears to be phylogenetically localized in a small number of Lepidoptera groups whose relationships remain unresolved. Beyond pinpointing these organisms’ taxonomic placement, it is likely that a better sampling of genomic data and a more systematic adducement of larval characters will contribute to our understanding of tribal and subfamilial boundaries. Likewise, the non-random distribution of recorded fern hosts across noctuid genera raises a number of specific, testable questions surrounding the evolution of diet breadth, and specifically the origins of pteridivory as perhaps a derived outcome of detritivory, moss- and lichen-eating.

## Figures

**Figures 1–6.**
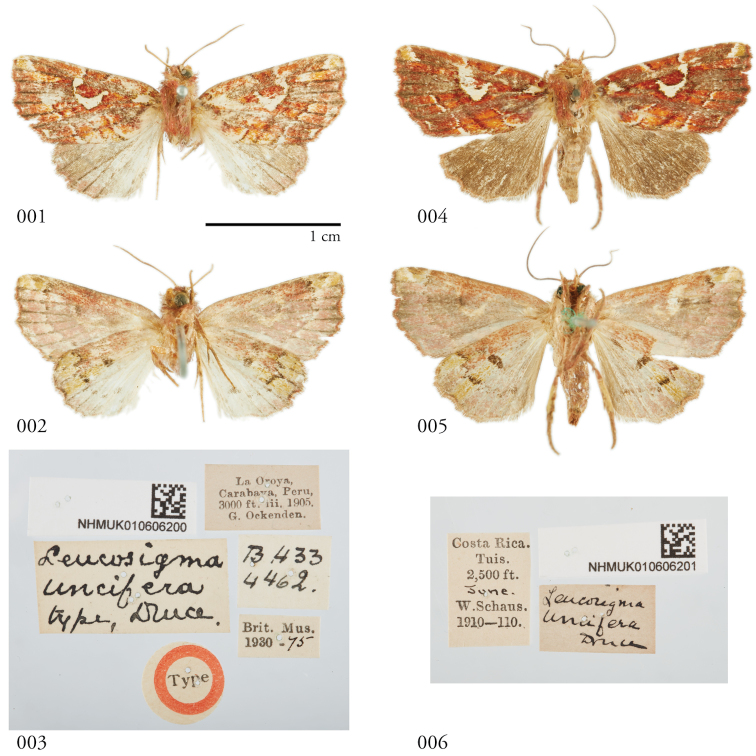
Dorsal and ventral habitus and labels of of *Leucosigmauncifera*. **(1–3)** Holotype ♂, Peru, NHMUK010606200 **(4–6) 4** Female, Costa Rica, NHMUK010606201.

**Figures 7–12.**
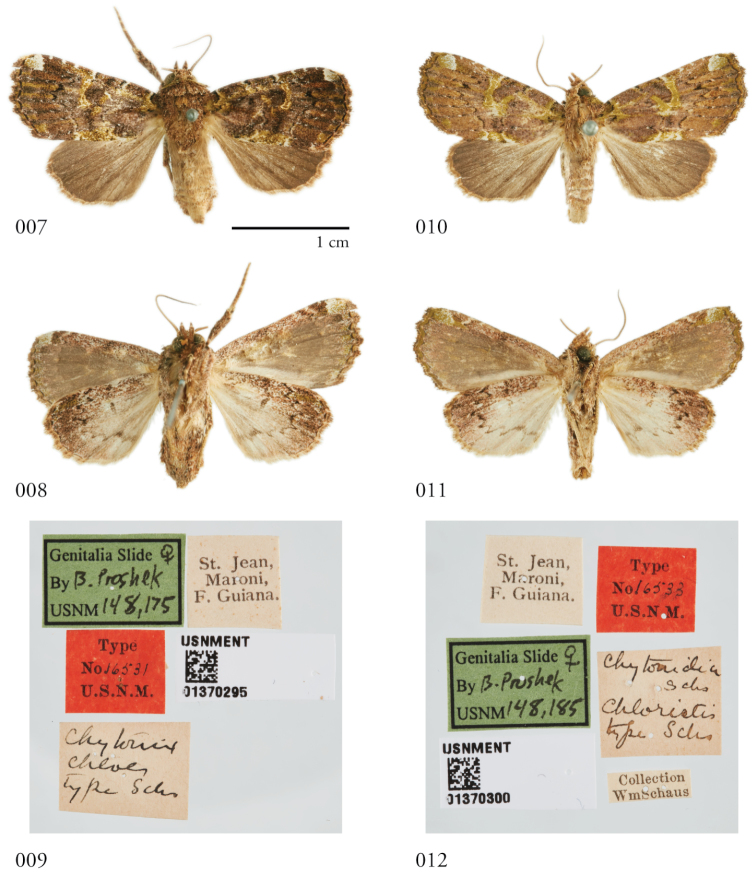
Dorsal and ventral habitus and specimen labels of female holotypes of *Chytonixchloe***(7–9)** and its synonym *Chytonidiachloristis***(10–12)**.

**Figures 13–22.**
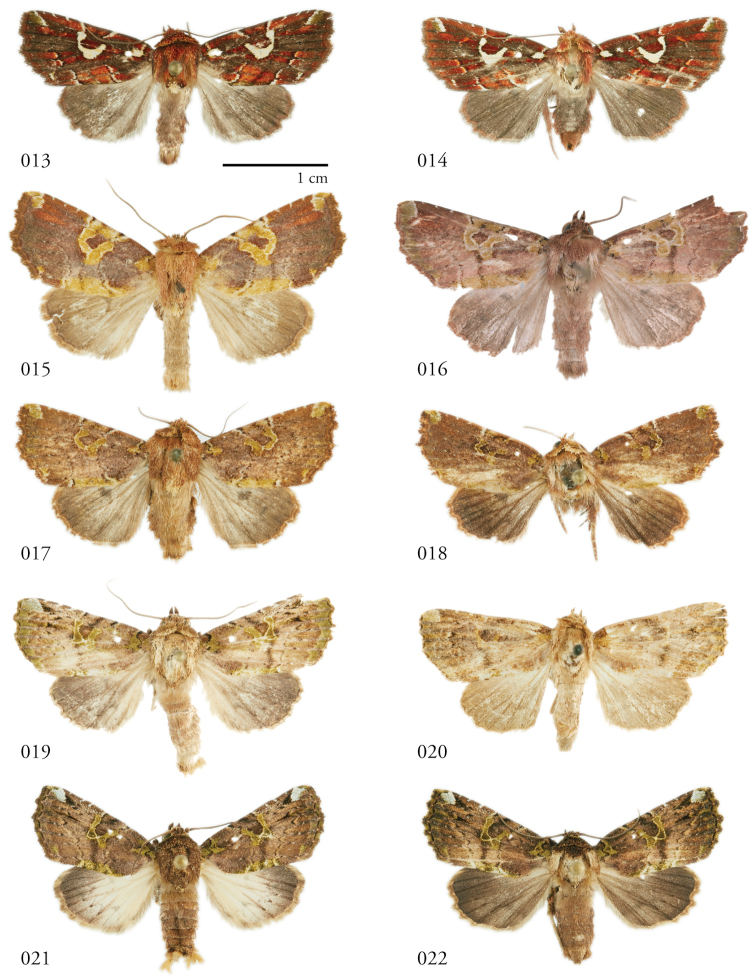
*Leucosigma* dorsal habitus. **13***L.uncifera* ♂, Área de Conservación Guanacaste (ACG), Costa Rica, 07-SRNP-109451, USNMENT01370293, USNM Dissection 148101 **14***L.uncifera* ♀, ACG, 12-SRNP-105657, USNMENT01370289 **15***L.solisae* ♂, Peru, NHMUK 010606203 **16***L.solisae* ♂ Holotype, ACG, 07-SRNP-101229, USNMENT01370294, USNM Dissection 148077 **17***L.poolei* ♂, Turrialba, Costa Rica, USNMENT01370290, USNM Dissection 148147, **18***L.poolei* Holotype ♀, ACG, 10-SRNP-70737, USNMENT01370296, USNM Dissection 148073 **19***L.reletiva* ♂, ACG, 09-SRNP-107116, USNMENT01370297, USNM Dissection 148177 ♂ **20***L.reletiva* Holotype ♀, Panama, USNMENT00973166, USNM Dissection 148170 **21***L.chloe* ♂, ACG, 96-SRNP-11371, USNMENT01370286 **22***L.chloe* ♀, ACG, 96-SRNP-11374, USNMENT01370299.

**Figures 23–32.**
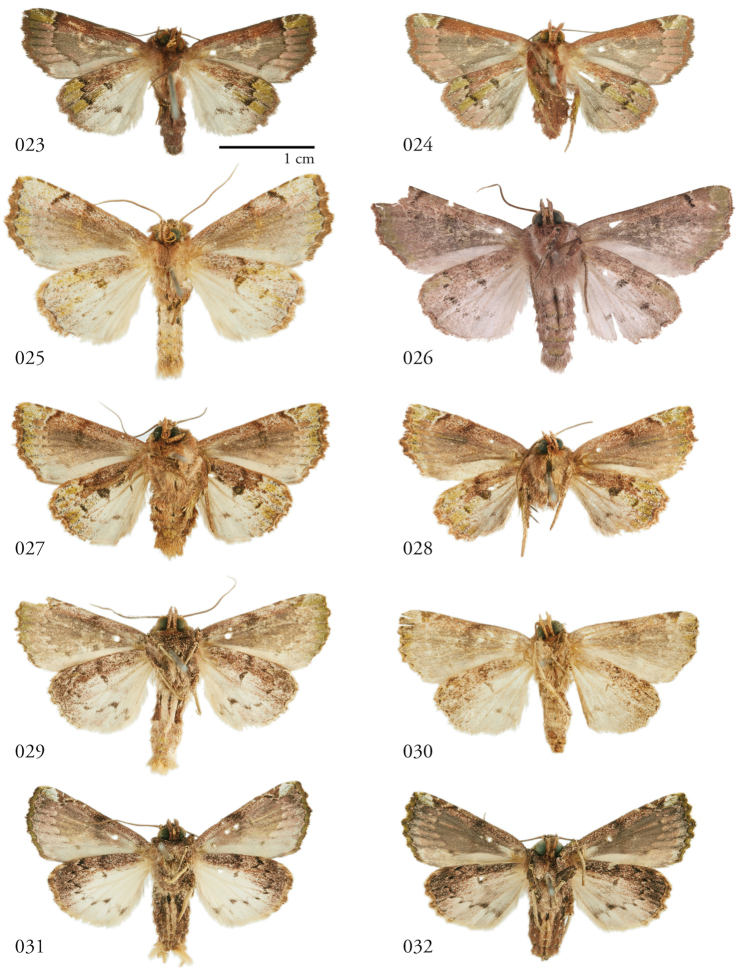
*Leucosigma* ventral habitus. **23***L.uncifera* ♂, Área de Conservación Guanacaste (ACG), Costa Rica, 07-SRNP-109451, USNMENT01370293, USNM Dissection 148101 ♂ **24***L.uncifera* ♀, ACG, 12-SRNP-105657, USNMENT01370289 **25***L.solisae* ♂, Peru, NHMUK 010606203 **26***L.solisae* Holotype ♂, ACG, 07-SRNP-101229, USNMENT01370294, USNM Dissection 148077 **27***L.poolei* ♂, Turrialba, Costa Rica, USNMENT01370290, USNM Dissection 148147 **28***L.poolei* Holotype ♀, ACG, 10-SRNP-70737, USNMENT01370296, USNM Dissection 148073 **29***L.reletiva* ♂, ACG, 09-SRNP-107116, USNMENT01370297, USNM Dissection 148177 **30***L.reletiva* ♀ Holotype, Panama, USNMENT00973166, USNM Dissection 148170 **31***L.chloe* ♂, ACG, 96-SRNP-11371, USNMENT01370286 **32***L.chloe* ♀, ACG, 96-SRNP-11374, USNMENT01370299.

**Figures 33–42.**
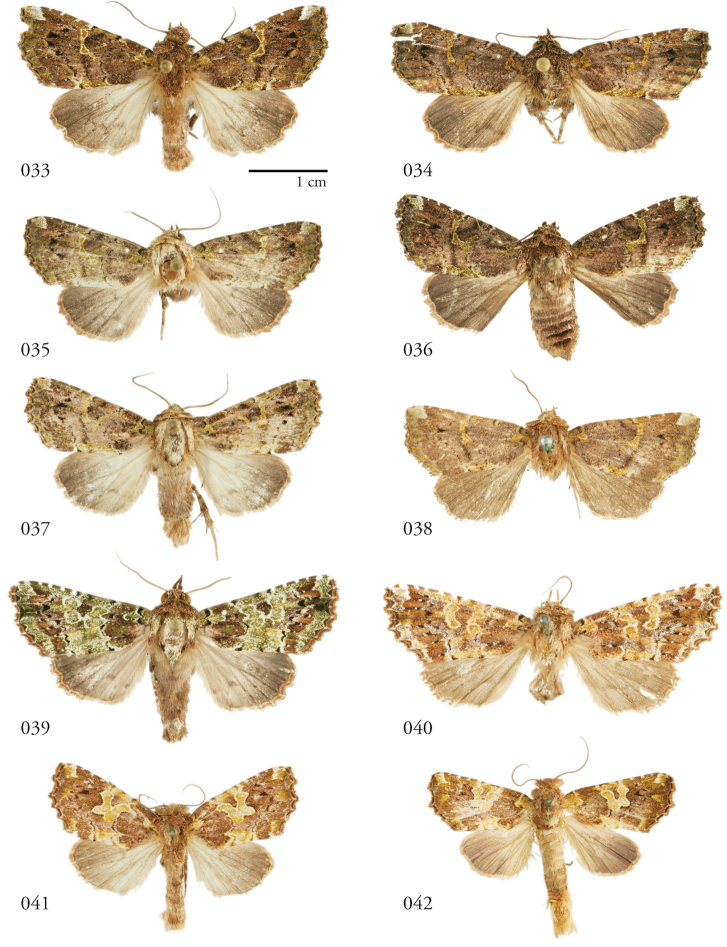
*Leucosigma* dorsal habitus. **33**L.sp.nr.schausi, ♂, Dominican Republic, USNMENT01370285, USNM Dissection 148142 **34**L.sp.nr.schausi,♀, Área de Conservación Guanacaste (ACG), Costa Rica, 11-SRNP-44977, USNMENT01370288, USNM Dissection 148079 **35***L.schausi* Holotype ♂, ACG, 10-SRNP-72230, USNMENT01370303, USNM Dissection 148076 **36***L.schausi* ♀, ACG, 10-SRNP-70113, USNMENT01370292 **37***L.schausi* ♂, Costa Rica, USNMENT01370291, USNM Dissection 148146 **38***L.schausi* ♀, Cuba, genitalia Slide USNM Noc 4092, USNMENT01370281 **39***L.albimixta* ♂, ACG, 10-SRNP-104564, USNMENT01370298 **40***L.albimixta* Holotype ♀, Costa Rica, USNMENT01370283, USNM Dissection 148184 **41***L.viridipicta* ♂, Peru, NHMUK010606202 **42***L.viridipicta* Holotype ♂, French Guiana, USNMENT 00973419, USNM Dissection 148176.

**Figures 43–52.**
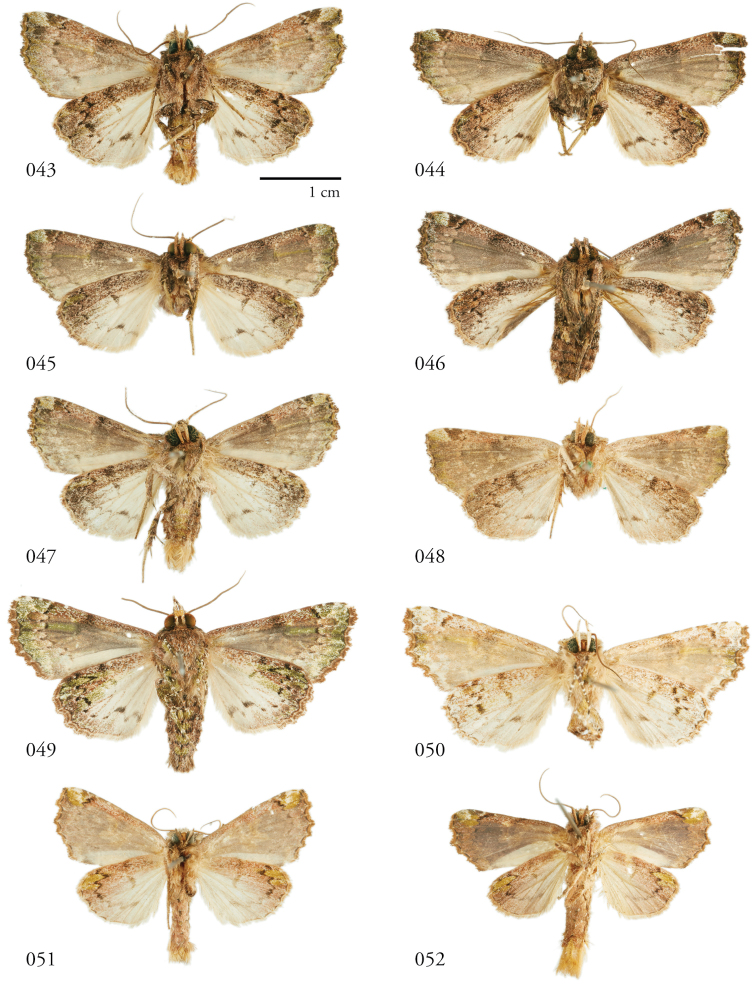
*Leucosigma* ventral habitus. **43**L.sp.nr.schausi, ♂, Dominican Republic, USNMENT01370285, USNM Dissection 148142 **44**L.sp.nr.schausi,♀, Área de Conservación Guanacaste (ACG), Costa Rica, 11-SRNP-44977, USNMENT01370288, USNM Dissection 148079 **45***L.schausi* ♂, ACG, 10-SRNP-72230, USNMENT01370303, USNM Dissection 148076 **46***L.schausi* ♀, ACG, 10-SRNP-70113, USNMENT01370292 **47***L.schausi* ♂, Costa Rica, USNMENT01370291, USNM Dissection 148146 **48***L.schausi* ♀, Cuba, genitalia Slide USNM Noc 4092, USNMENT01370281 **49***L.albimixta* ♂, ACG, 10-SRNP-104564, USNMENT01370298 **50***L.albimixta* Holotype ♀, Costa Rica, USNMENT01370283, USNM Dissection 148184 **51***L.viridipicta* ♂, Peru, NHMUK 010606202 **52***L.viridipicta* Holotype ♂, French Guiana, USNMENT 00973419, USNM Dissection 148176.

**Figures 53–60.**
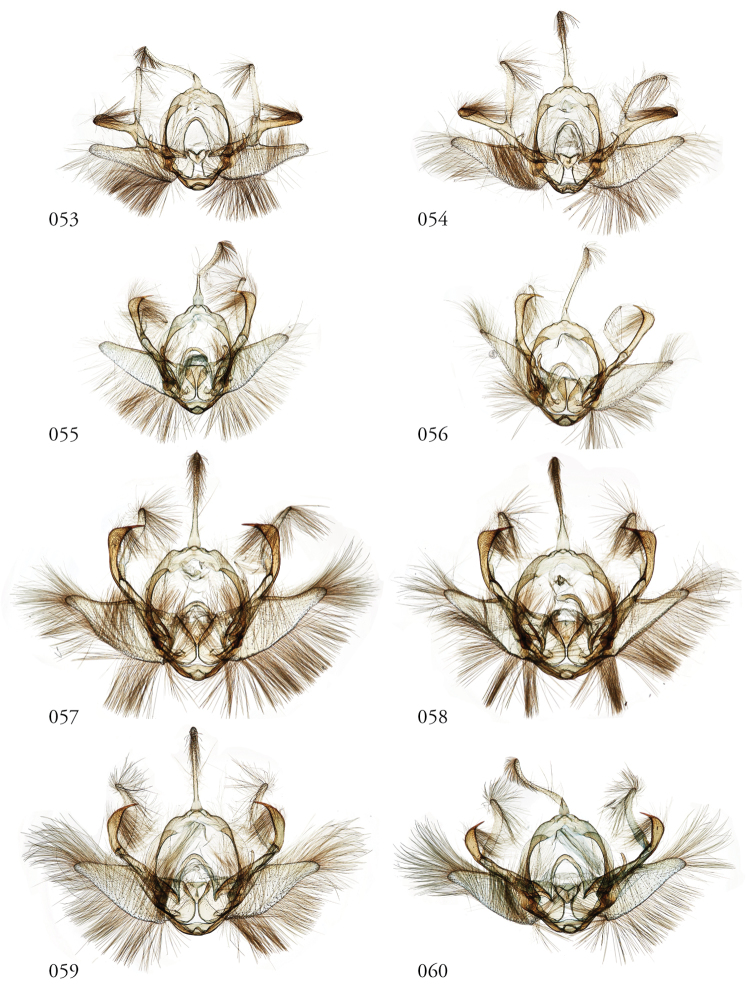
*Leucosigma* male genitalia, valves. **53***L.uncifera* Holotype, Peru, NHMUK010606200 **54***L.uncifera*, Área de Conservación Guanacaste (ACG), Costa Rica, 06-SRNP-102374, USNMENT01438843, USNM Dissection 148199 **55***L.uncifera*, ACG, 07-SRNP-109451, USNMENT01370293, USNM Dissection 148101 **56***L.uncifera*, ACG, 11-SRNP-100029, USNMENT01438838, USNM Dissection 148102 **57***L.solisae* Holotype, ACG, 07-SRNP-101229, USNMENT01370294, USNM Dissection 148077 **58***L.solisae*, ACG, 07-SRNP-109201, USNMENT01437370, USNM Dissection 148078 **59***L.poolei*, Turrialba, Costa Rica, USNMENT01370290, USNM Dissection 148147 **60***L.poolei*, Turrialba, Costa Rica, USNMENT01438829, USNM Dissection 148148.

**Figures 61–68.**
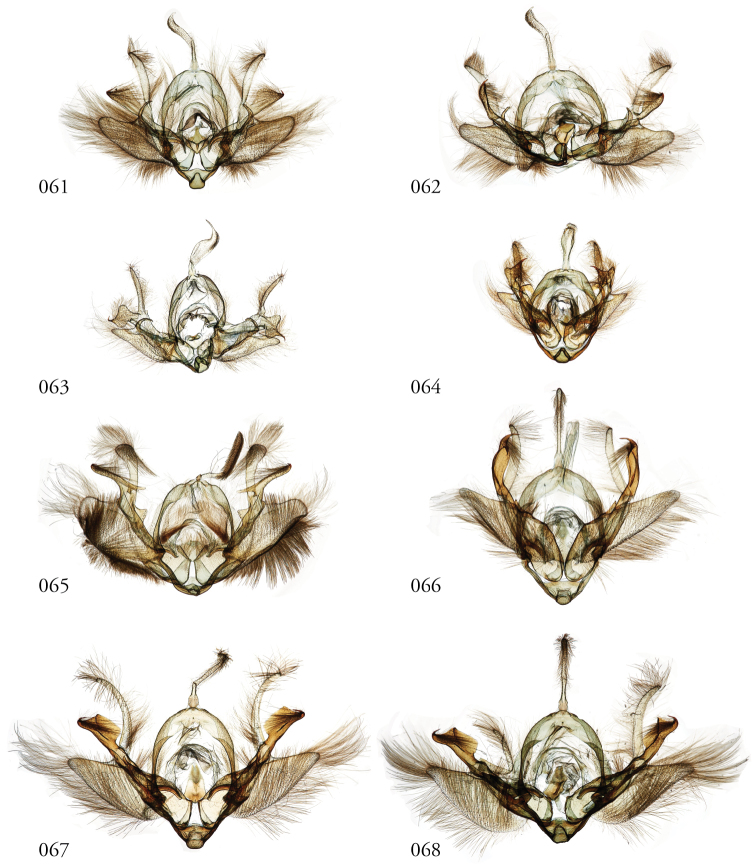
*Leucosigma* male genitalia, valves. **61***L.reletiva*, Área de Conservación Guanacaste (ACG), Costa Rica, 07-SRNP-101206, USNMENT01437211, USNM Dissection 148178 **62***L.reletiva*, ACG, 09-SRNP-107116, USNMENT01370297, USNM Dissection 148177 **63***L.chloe*, ACG, 07-SRNP-108955, USNMENT01437367, USNM Dissection 148055 **64***L.chloe* ♂, ACG, 96-SRNP-11375, USNMENT01438814, USNM Dissection 148070 **65***L.viridipicta* Holotype, French Guiana, USNMENT00973419, USNM Dissection 148176 **66***L.albimixta*, ACG, 11-SRNP-30511, USNM 00105321, USNM Dissection 148085 **67***L.albimixta*, ACG, 07-SRNP-102199, USNMENT01437365, USNM Dissection 148068 **68***L.albimixta* 10-RNSP-107587, USNMENT01437230, USNM Dissection 148069.

**Figures 69–72.**
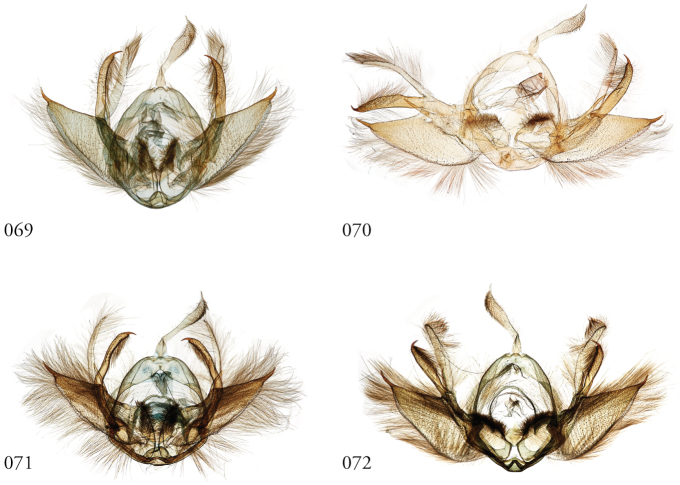
*Leucosigmaschausi*, male genitalia, valves. **69** Área de Conservación Guanacaste (ACG), Costa Rica,148298 10-SRNP-70740, USNMENT01437325 **70** USNMENT01437241, USNM Dissection 4091 **71** Costa Rica, USNMENT01370291, USNM Dissection 148146 **72**ACG, 10-SRNP-72230, USNMENT01370303, USNM Dissection 148076.

**Figures 73–76.**
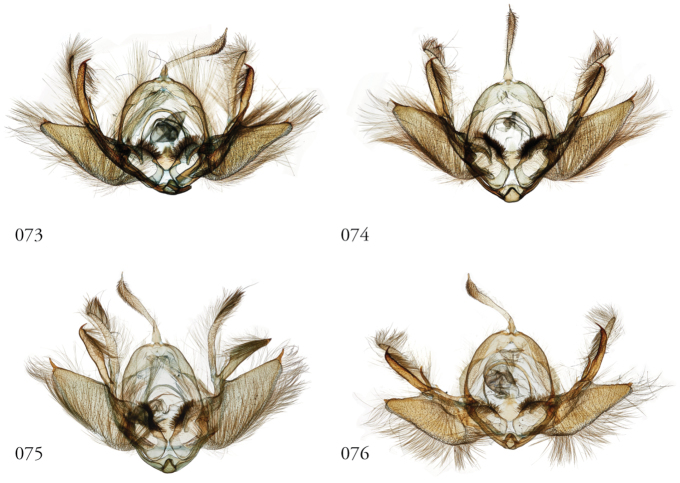
Leucosigmasp. nearschausi, male genitalia, valves. **73** Dominican Republic, USNMENT01370285, USNM Dissection 148142 **74** Área de Conservación Guanacaste (ACG), Costa Rica, 11-SRNP-69183, USNMENT01438819, USNM Dissection 148075 **75**ACG, 10-SRNP-73224, USNMENT00105325, USNM Dissection 148296 **76** Dominican Republic, USNMENT01437231, USNM Dissection 148297.

**Figures 77–84.**
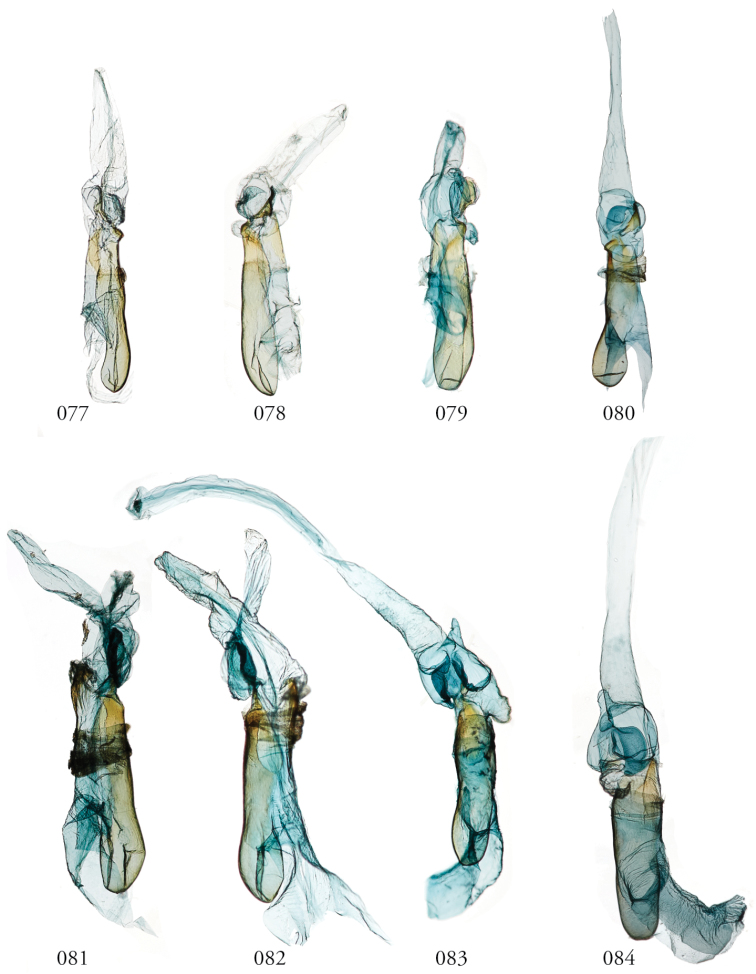
*Leucosigma* male genitalia, phalli. **77***L.uncifera*, Holotype, Peru, NHMUK010606200 **78***L.uncifera*, Área de Conservación Guanacaste (ACG), Costa Rica, 06-SRNP-102374, USNMENT01438843, USNM Dissection 148199 **79***L.uncifera*, ACG, 07-SRNP-109451, USNMENT01370293, USNM Dissection 148101**80***L.uncifera*, USNMENT01437285, USNM Dissection 148302 **81***L.solisae*, ACG, 07-SRNP-109201, USNMENT01437370, USNM Dissection 148078 **82***L.solisae*, ACG, 07-SRNP-101229,USNMENT01370294, USNM Dissection 148077 **83***L.poolei*, Turrialba, Costa Rica,USNMENT01438829, USNM Dissection 148148 **84***L.poolei*, Turrialba, Costa Rica, USNMENT01370290, USNM Dissection 148147.

**Figures 93–95.**
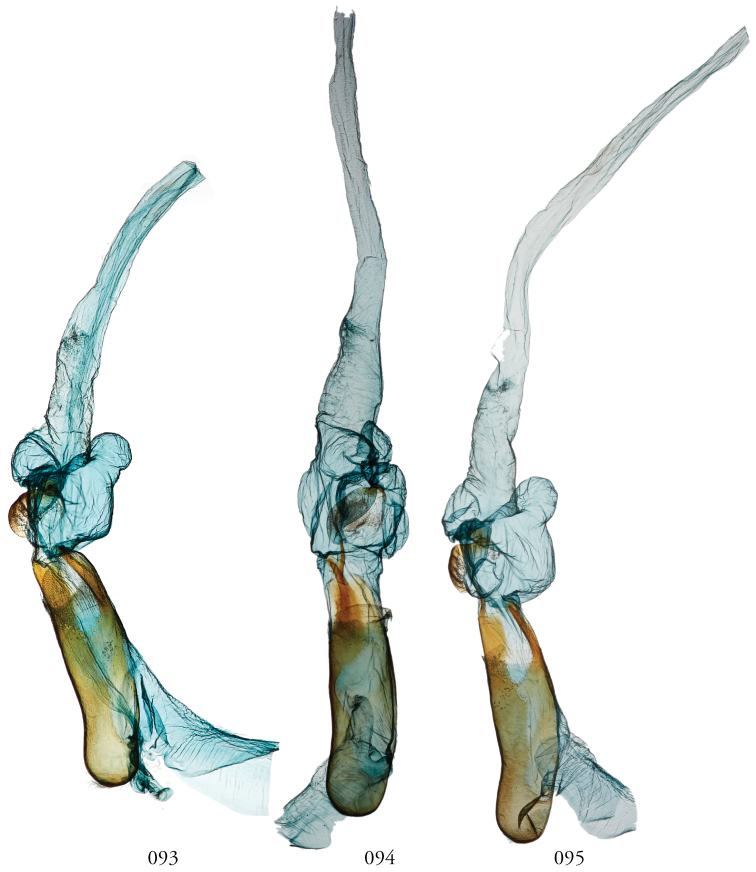
Leucosigmasp. nearschausi, male genitalia, phalli. **93** Dominican Republic, USNM Dissection 148142, USNMENT01370285 **94** USNMENT00105325, USNM Dissection 148296 **95** Dominican Republic, USNMENT01437231, USNM Dissection 148297.

**Figures 96–98.**
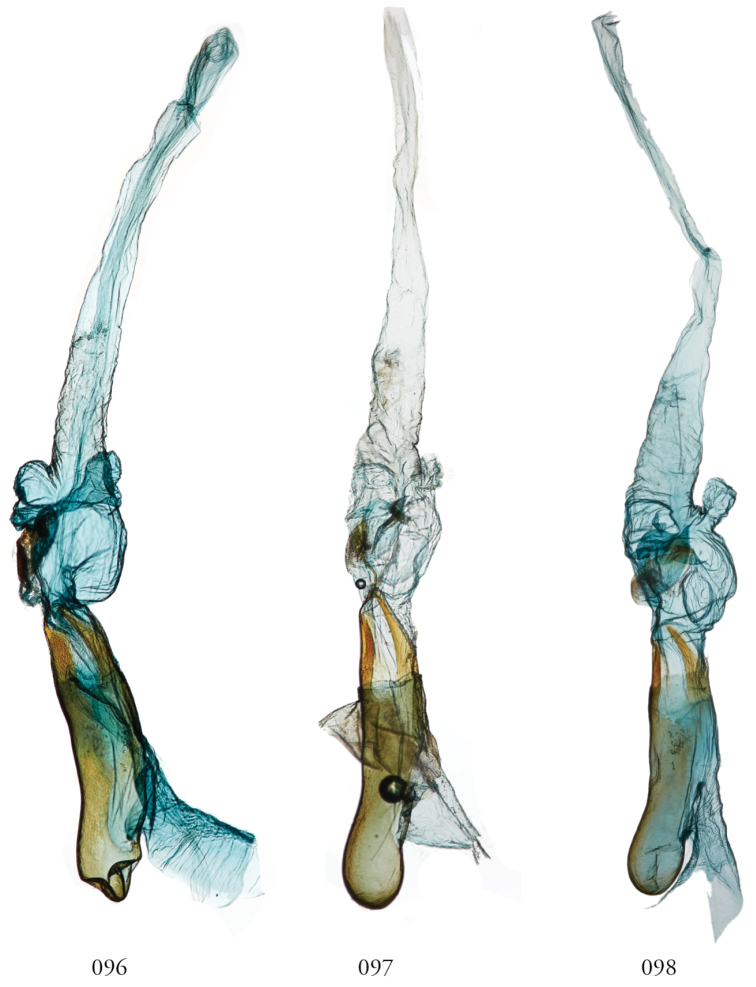
*Leucosigmaschausi*, male genitalia, phalli. **96** Costa Rica, USNMENT01370291, USNM Dissection 148146 **97** Área de Conservación Guanacaste (ACG), Costa Rica, 10-SRNP-72230, USNMENT01370303, USNM Dissection 148076 **98** USNMENT01437325, USNM Dissection 148298.

**Figures 99–104.**
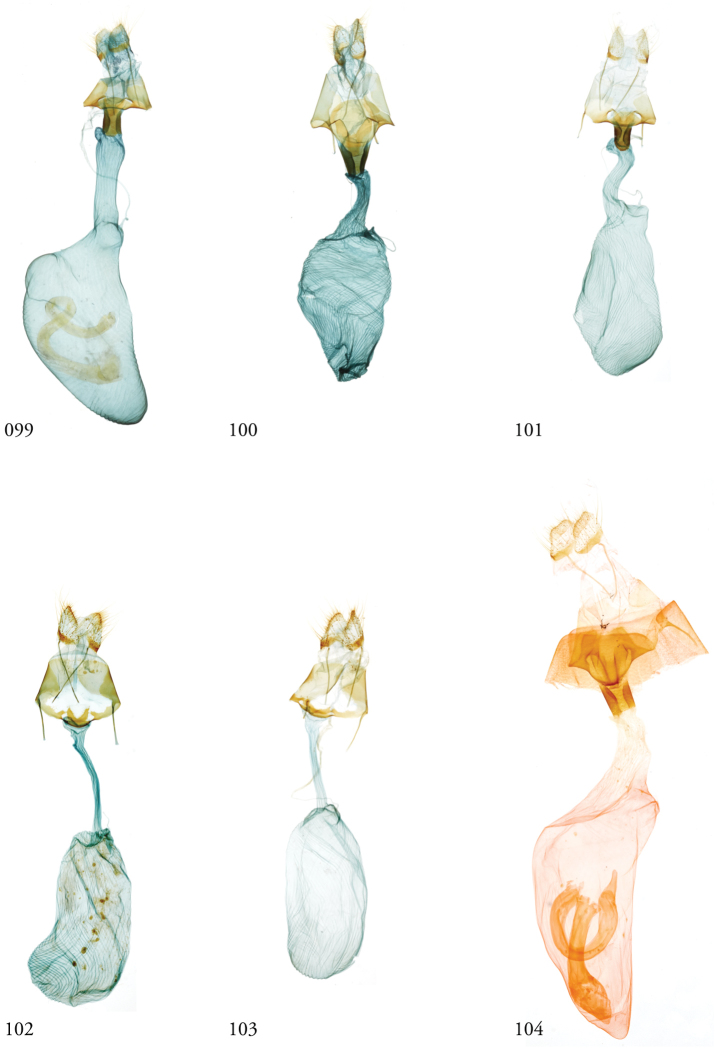
*Leucosigma* female genitalia. **99***L.chloe*, Brazil, USNMENT01437322, USNM Dissection 148152 **100***Chytonixchloe* (= *L.chloe*) Holotype USNMENT01370295, USNM Dissection 148175 **101***Chytonidiachloristis* Holotype (= *chloe*) USNMENT01370300 USNM Dissection 148185 **102***L.poolei*, Área de Conservación Guanacaste (ACG), Costa Rica, 10-SRNP-70737, USNM Dissection 148073, USNMENT01370296 **103***L.poolei*USNM Dissection 148074 **104***L.schausi*, Cuba, USNMENT01370281, USNM Noc 4092 (cf. 4093).

**Figures 105–110.**
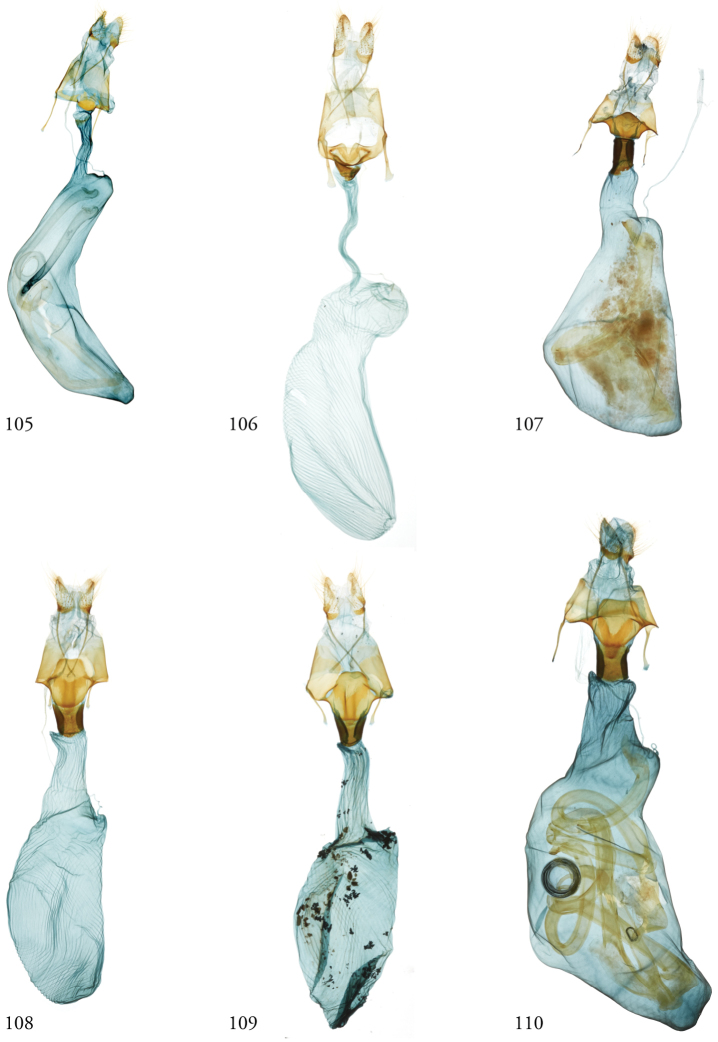
*Leucosigma* female genitalia. **105***L.uncifera*, Área de Conservación Guanacaste (ACG), Costa Rica, 11-SRNP-102284, USNMENT01437255, USNM Dissection 148104 **106***L.albimixta* Holotype, Costa Rica, USNMENT01370283, USNM Dissection 148184 **107***L.chloe*, ACG, USNMENT01437380, USNM Dissection 148169 **108***L.schausi*USNM Dissection 148080 **109**L.sp. nearschausi, ACG, 11-SRNP-44977, USNMENT01370288, USNM Dissection 148079 **110***L.reletiva* Holotype, Panama, USNMENT00973166, USNM Dissection 148170.

**Figures 129–130.**
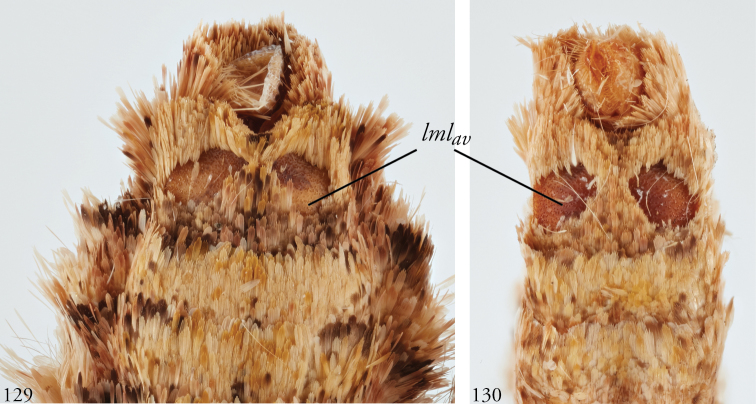
Female abdomen, ventral aspect, illustrating paired sternal sclerotizations in two specimens of the *Leucosigmachloe* complex. **129** MEX: Tmps Gomez Farias 21 III 1981, Nacimiento del Rio Frio, Gillespy & Lara Collectors; USNMENT01437352 **130** Bocas dToro Pan, Apr ‘07, Collection Wm Schaus; USNMENT01437300.

## Supplementary Material

XML Treatment for Leucosigma

XML Treatment for Leucosigma uncifera

XML Treatment for Leucosigma reletiva

XML Treatment for Leucosigma albimixta

XML Treatment for Leucosigma chloe

XML Treatment for Leucosigma viridipicta

XML Treatment for Leucosigma solisae

XML Treatment for Leucosigma poolei

XML Treatment for Leucosigma schausi
